# Longitudinal dynamics between other-gender peer contact quality and gender-stereotypical trait norms in coeducational physical education: a four-wave random-intercept cross-lagged panel study

**DOI:** 10.3389/fpsyg.2026.1923110

**Published:** 2026-09-04

**Authors:** Dongyu Liu, Xianlin Xiao, Xiaochuan Hang

**Affiliations:** 1 School of Physical Education, Southwest Petroleum University, Chengdu, China; 2 Faculty of Management, Shinawatra University, Pathum Thani, Thailand; 3 Department of Physical Education, Nanjing Medical University, Nanjing, China

**Keywords:** coeducational physical education, gender socialization, gender-stereotypical trait norms, longitudinal study, other-gender peer contact, RI-CLPM

## Abstract

Coeducational physical education (PE) may reproduce or challenge gender norms depending on the quality of students’ interactions with other-gender peers. This four-wave longitudinal study examined between-person and within-person prospective associations between other-gender peer contact quality in PE (CQ) and endorsement of gender-stereotypical trait norms (GST). Participants were 1,376 students from 20 primary and secondary schools in Chengdu, China, assessed from March 2025 to January 2026. After longitudinal measurement invariance was established, a random-intercept cross-lagged panel model separated stable between-person differences from time-specific within-person deviations. The CQ and GST random intercepts were negatively correlated (*r* = −0.287, *p* < 0.001). In the freely estimated model, higher-than-usual CQ was associated with lower-than-usual GST at the next wave (*β* = −0.197, −0.133, and −0.151; all *p* ≤ 0.001), whereas the reverse paths were nonsignificant (*β* = 0.013, 0.001, and 0.030). Equality constraints across intervals were compatible with the data, scaled Δχ^2^(8) = 1.869, *p* = 0.985. Direct Wald tests showed that the CQ-to-GST paths were more negative than the corresponding reverse paths, χ^2^(3) = 44.574, *p* < 0.001. The pattern remained in a 44-indicator multiple-indicator RI-CLPM and after removal of the CQ equality item. Full metric and scalar invariance held across boys and girls, and no statistically detectable sex-group differences emerged in either cross-lagged direction. The findings indicate a temporally asymmetric prospective association in the pooled sample. Causal, developmental-stage, and classroom-level interpretations remain limited by the observational design and unavailable age, grade, and clustering identifiers.

## 1 Introduction

Gender norms are learned through repeated social interactions as well as explicit instruction. During late childhood and early adolescence, expectations concerning toughness, emotional restraint, modesty, protection, leadership, and appropriate social behavior become increasingly salient, while peer approval and school experiences gain influence alongside family socialization (Kågesten et al., 2016). These norms can shape interpersonal relationships, educational choices, participation in physical activity, and willingness to enter gender-atypical roles (Blum et al., 2017; Heise et al., 2019; Mmari et al., 2017).

Coeducational physical education (PE) is a distinctive setting for examining this process because gendered competencies and roles are enacted publicly and repeatedly. Physical performance, competition, risk-taking, leadership, help-seeking, emotional reactions, and dependence on teammates are visible to peers. Teachers also organize groups, distribute opportunities, select activities, and authorize particular forms of participation. PE therefore combines embodied performance, repeated task interdependence, and institutional regulation in a way that can either reinforce familiar gender hierarchies or expose students to credible counter-stereotypical behavior.

The central issue is not simply whether boys and girls occupy the same class. Students can experience the same nominally mixed-gender lesson as cooperative and equal, or as exclusionary and hierarchical. The present study defines other-gender peer contact quality in PE (CQ) as the perceived closeness, cooperation, equality, and pleasantness of interactions with classmates of a different gender. It examines CQ in relation to gender-stereotypical trait norms (GST), defined as endorsement of prescriptive beliefs linking boys with toughness, emotional restraint, and self-protection and girls with modesty, quietness, and greater need for protection. An integrated account must explain both how contact experiences may precede later norm endorsement and how prior norms may shape subsequent interaction quality.

### 1.1 Coeducational physical education as an embodied and institutionally organized contact context

Gender-stereotypical trait norms prescribe how boys and girls should behave and identify the social consequences of violating those expectations (Koenig, 2018). Cognitive theories of gender development propose that gender schemas guide attention, memory, evaluation, and behavior once gender categories become salient (Martin et al., 2002). Developmental intergroup theory adds that institutional practices can make social categories appear functionally meaningful, encouraging children to associate group membership with particular attributes and roles (Bigler and Liben, 2007). PE is especially relevant because its activities repeatedly make gender categories, competence judgments, and role expectations observable.

In PE, students see who is trusted with leadership, who controls equipment or the ball, whose mistakes are tolerated, who is expected to take physical risks, and who is protected from challenge. Unequal opportunities and gender-typed task assignments can confirm traditional expectations; varied roles, shared responsibility, and visible counter-stereotypical competence can challenge them. Research has therefore cautioned that coeducation should not be equated with gender integration. Mixed-gender participation can preserve hierarchy when a small number of students dominate play or when leadership and support roles remain gender-typed (Cárcamo et al., 2021; Fagrell et al., 2012; Frühauf et al., 2022; Hills and Croston, 2012).

This distinction makes contact quality more informative than contact quantity or co-presence. Two students may spend comparable time in mixed-gender groups while experiencing fundamentally different status relations, cooperation, and emotional climates. A recent review of gendered intergroup contact similarly concluded that contact between genders is common but varies substantially in quality, content, intimacy, status, and voluntariness (Mikołajczak et al., 2025). The relevant developmental exposure may therefore be what occurs within recurring contact rather than the formal presence of another gender.

The four CQ dimensions are complementary. Closeness and pleasantness capture relational and affective tone, cooperation captures task interdependence, and equality captures perceived status relations. Together they correspond to central conditions in intergroup contact theory and to pedagogical features emphasized in research on equitable coeducational PE, including shared goals, role rotation, equal participation, and teacher support (Brown and Hewstone, 2005; Dovidio et al., 2017; Pettigrew and Tropp, 2006, 2008).

CQ is an interpersonal experience embedded within a broader multilevel PE ecology. Students’ perceptions of cooperation, equality, and respect may reflect teacher grouping and feedback, pedagogical organization, activity selection, access to space and equipment, and wider school norms. Reviews of physical-activity interventions in children and adolescents show that individual and interpersonal determinants have received substantially more attention than organizational and environmental determinants, while effects on targeted determinants and behavior have generally been limited or uncertain (Ciaccioni et al., 2026; Khudair et al., 2025; Kolovelonis et al., 2024; Ling et al., 2024). Their broader implication is that peer-contact quality is best considered alongside teacher, organizational, and environmental conditions.

### 1.2 An integrated contact–schema account of CQ and GST

Intergroup contact theory provides the main basis for expecting CQ to precede subsequent GST. Contact is most consistently associated with favorable intergroup outcomes when it includes equal status, cooperation, common goals, and institutional support (Pettigrew and Tropp, 2006). Proposed mediating processes include reduced anxiety, greater empathy and perspective taking, and exposure to individuating information that contradicts simplified group beliefs (Pettigrew and Tropp, 2008). These mechanisms are relevant to gender because gender categories organize expectations, identities, and patterns of interaction.

PE gives these processes a concrete behavioral form. Equal participation can expose students to competence that conflicts with traditional expectations; interdependent tasks make other-gender classmates’ contributions consequential; repeated positive exchanges may build trust; and teacher-supported equality can signal that non-stereotypical roles are legitimate. A girl leading tactical decisions or a boy offering emotional support supplies observable information that is difficult to reconcile with rigid trait–gender prescriptions.

Longitudinal research offers partial support for this contact-socialization account. Children who gained other-gender friendships subsequently reported more positive gender attitudes (Halim et al., 2021), and network evidence suggests that other-gender friendships can precede more egalitarian gender-role attitudes under some conditions (Kretschmer, 2024). Other work has linked the quality of gender-based peer interactions with later gender-related cognitions (Xiao et al., 2026). These studies differ in age, relationship type, and outcome, and few have examined recurring, teacher-structured contact in PE.

The reverse pathway is also theoretically plausible. Gender schemas shape expectations about competence, authority, toughness, protection, and appropriate participation (Martin et al., 2002). Stronger endorsement of GST may therefore influence role allocation, willingness to trust other-gender teammates, responses to mistakes, displays of dominance, avoidance of collaboration, and policing of gender-nonconforming behavior. Such actions could alter whether subsequent contact is cooperative and equal or strained and hierarchical.

PE changes the form of this reverse process. In voluntary friendship networks, attitudes may guide partner selection or avoidance. In regular PE, teachers and timetables often determine whether contact occurs and who works together, reducing students’ control over contact opportunities. Existing norms may still influence how students participate in and interpret assigned interactions, but they may have less leverage over whether those interactions occur. The reverse pathway is therefore best framed as norm enactment within an institutionally organized setting rather than as unrestricted contact selection.

Available evidence does not resolve the directional question. Some studies report reciprocal associations between gender attitudes and other-gender relationships, whereas others find limited evidence of attitude-based selection after relational processes are modeled (Halim et al., 2021; Kretschmer, 2024). Most studies also focus on friendship presence or composition rather than the subjective quality of recurring task-based interaction. Directly estimating both prospective directions is therefore necessary.

### 1.3 Empirical gaps and the need for a within-person longitudinal perspective

A stable association between CQ and GST can arise because students who generally experience better contact also generally endorse less stereotypical norms. That between-person relation is theoretically meaningful, but it does not show that a period of improved contact is followed by lower norm endorsement within the same student. Stable family socialization, personality, sibling experiences, school climate, or socioeconomic background could contribute to both constructs (Cislaghi and Heise, 2020; Mesman and Groeneveld, 2018).

The within-person question asks whether CQ above a student’s own usual level at one wave is associated with GST below that student’s usual level at the next wave. The reverse question asks whether a temporary elevation in GST is associated with lower subsequent CQ relative to the student’s own typical level. These prospective deviations answer a different question from stable differences between students.

Conventional cross-lagged panel models combine stable between-person and time-specific within-person variation when constructs contain trait-like variance (Hamaker et al., 2015). The random-intercept cross-lagged panel model (RI-CLPM) separates these components: random intercepts represent stable individual differences, while lagged paths among within-person factors represent prospective associations between temporary deviations (Hamaker et al., 2015; Usami, 2021). This decomposition improves level-specific interpretation but does not remove time-varying confounding or establish causal effects.

The distinction is substantively important. In other intergroup settings, longitudinal contact–attitude associations have sometimes been located primarily at the between-person level once random intercepts are introduced (Friehs et al., 2024). Gender-contact research has consequently called for models that separate stable differences from within-person prospective processes and test both directions over time (Mikołajczak et al., 2025).

Three empirical gaps remain. Research rarely examines repeated, task-based other-gender contact quality in PE; many longitudinal studies use only two waves and cannot evaluate whether estimates are compatible with temporal equality; and Chinese evidence on gender-stereotypical trait norms remains largely cross-sectional or outside PE (Moreau et al., 2021; Yu et al., 2022). Four waves permit freely estimated paths across three transitions, formal comparison with equality-constrained models, and direct tests of whether the two prospective directions differ.

The study therefore integrates contact theory, gender-schema accounts, and the institutional organization of PE within one framework. It distinguishes stable CQ–GST covariation, a prospective CQ-to-GST pathway consistent with contact socialization, and a prospective GST-to-CQ pathway consistent with norm enactment.

### 1.4 The present study

The present study examined the longitudinal association between CQ and GST across four waves among students attending primary and secondary schools in Chengdu, China. A bivariate RI-CLPM separated stable between-person differences from within-person deviations and estimated autoregressive, concurrent, and reciprocal cross-lagged associations. Freely estimated paths were reported before evaluating whether lagged coefficients could be treated as equal across the three observed transitions.

Hypothesis 1 (H1) proposed a negative between-person association: students with characteristically higher CQ would show lower characteristic endorsement of GST, reflected in a negative correlation between the two random intercepts.

Hypothesis 2 (H2) proposed a negative CQ-to-GST within-person pathway: CQ above a student’s usual level at one wave would be associated with GST below that student’s usual level at the subsequent wave.

Hypothesis 3 (H3) proposed a negative reverse pathway: GST above a student’s usual level at one wave would be associated with CQ below that student’s usual level at the subsequent wave.

**Figure 1:**
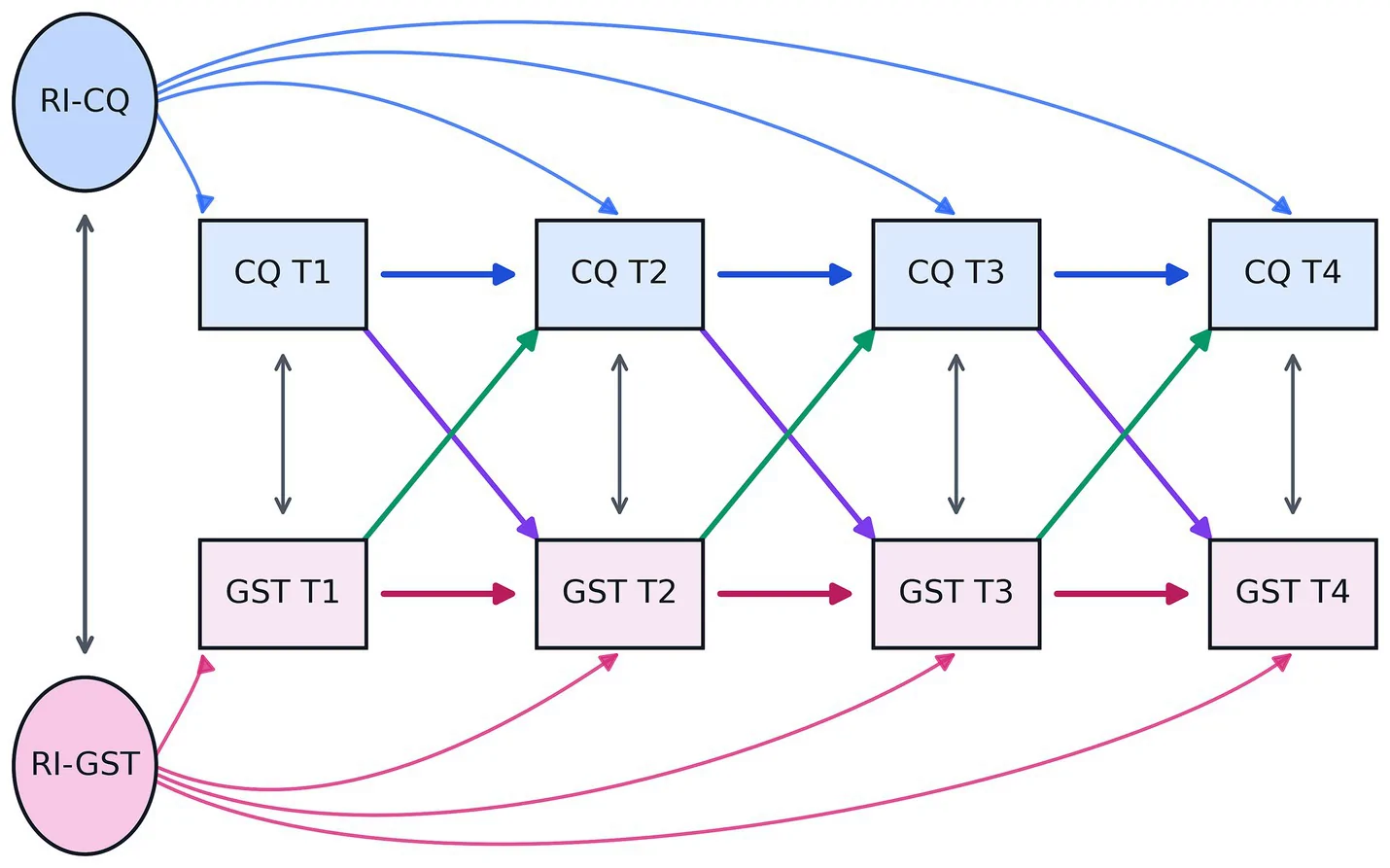
Hypothesized four-wave random-intercept cross-lagged panel model of other-gender peer contact quality and gender-stereotypical trait norms. CQ, other-gender peer contact quality; GST, gender-stereotypical trait norms; RI, random intercept; T1–T4, Waves 1–4. Single-headed horizontal arrows represent autoregressive paths, and diagonal arrows represent reciprocal cross-lagged paths. Double-headed arrows represent the between-person correlation between the random intercepts and within-wave CQ–GST correlations. Residual variances and item-level indicators are omitted for visual clarity.

The reciprocal hypotheses did not assume equal magnitudes for the two directions. Figure 1 presents the hypothesized panel model, in which both prospective pathways are estimated directly.

## 2 Materials and methods

### 2.1 Participants and procedure

This study used a four-wave longitudinal panel design to examine prospective within-person associations between other-gender peer contact quality and gender-stereotypical trait norms in coeducational PE. Participants were recruited from 20 primary and secondary schools in Chengdu, Sichuan Province, China. All participating students were minors and attended regular coeducational PE classes during the study period. Individual age, grade, student-level school-stage information, and verified school, class, and PE-teacher identifiers were not retained in the analytic dataset and could not be reconstructed from the anonymous student codes. Developmental-group, cluster-robust, and multilevel analyses were therefore unavailable, and empirical claims are restricted to the pooled sample.

Data collection began in March 2025 and was conducted during regular school terms. The four assessment waves were arranged to capture theoretically meaningful changes in students’ peer-contact experiences and gender-related norms while minimizing excessive recall burden and disruption from the school calendar. Data were collected at T1 from March 17 to March 28, 2025; at T2 from May 26 to June 6, 2025; at T3 from October 20 to October 31, 2025; and at T4 from January 5 to January 16, 2026. Although the calendar intervals were not identical because of the summer vacation, each adjacent wave represented approximately 8–10 effective school weeks of exposure to regular physical education. This spacing was selected because the focal processes were expected to unfold through repeated physical education experiences rather than through isolated lessons. In longitudinal research, the temporal design should be aligned with the theoretical pace of change, and cross-lagged effects may vary as a function of the measurement interval (Collins, 2006; Dormann and Griffin, 2015). The summer vacation was not treated as an equivalent exposure period because students did not receive regular school-based physical education during that time. Therefore, T3 was administered after students had resumed regular physical education classes for several weeks in the autumn semester.

The study protocol was reviewed and approved by the Ethics Committee of the Department of Physical Education, Nanjing Medical University, China. Because all participants were minors, written informed consent was obtained from a parent or legal guardian, and written assent was obtained from each student before data collection. Students were informed that participation was voluntary, that they could withdraw at any time without penalty, and that their responses would be used only for research purposes. To protect confidentiality, no student names were recorded on the questionnaires. Instead, each participant used a unique non-identifying code to match responses across waves. Individual responses were not disclosed to teachers, parents, or school administrators (Council for International Organizations of Medical Sciences, 2016).

Questionnaires were administered offline in classrooms by trained research assistants using standardized instructions. Teachers helped maintain normal classroom order when necessary but did not inspect students’ responses. Students completed the paper-and-pencil questionnaires independently during regular school time. At baseline, 1,671 questionnaire packets were distributed and 1,489 were returned, yielding a response rate of 89.11%. After invalid questionnaires were excluded, 1,376 valid baseline questionnaires remained, corresponding to 92.41% of returned questionnaires and an overall valid response rate of 82.35%. The baseline sample consisted of 712 boys (51.74%) and 664 girls (48.26%). The baseline demographic characteristics are presented in Table 1.

**Table 1:** Baseline demographic characteristics of participants.

Variable	Category	*n*	%
Sex	M (Male)	712	51.74
Sex	F (Female)	664	48.26
Residence	U (Urban)	894	64.97
Residence	R (Rural)	482	35.03
Sibling configuration	OC (Only child)	699	50.80
Sibling configuration	SS (Same-sex siblings only)	212	15.41
Sibling configuration	MS (Mixed-sex siblings)	465	33.79
Highest parental education	L (Junior high school or below)	384	27.91
Highest parental education	M (High school or vocational school)	552	40.12
Highest parental education	H (College or above)	440	31.98

*N* = 1,376 at baseline. Percentages for demographic variables were calculated using the valid baseline sample as the denominator.

Questionnaires were screened according to predefined validity criteria. A questionnaire was considered invalid if it met any of the following conditions: absence of verifiable student assent or guardian consent documentation, more than 20% missing responses on substantive items, failure on the attention-check item, invariant or clearly patterned responses across almost all substantive items, or implausible demographic information. The attention-check item was: “To show that you are reading carefully, please choose ‘agree’ for this item.” This item was not included in any scale score. Across follow-up waves, questionnaires were additionally checked for code mismatches, duplicate codes, and impossible demographic changes. Cases with intermittent missingness were not automatically deleted from the longitudinal dataset; instead, the final analytic approach retained all available observations under the missing-data assumptions described in the data-analysis section.

The valid baseline sample was *N* = 1,376. Across the four waves, 1,073 students provided item-level responses at every assessment, representing 77.98% of the baseline sample. Accordingly, 303 students were missing or lost to follow-up at least once after baseline, representing 22.02% of the baseline sample. At the wave level, 1,376 students provided at least one item response at T1 (100.00%), 1,295 at T2 (94.11%), 1,233 at T3 (89.61%), and 1,202 at T4 (87.35%). Relative to baseline, the corresponding wave-level missingness or attrition was 81 students at T2 (5.89%), 143 students at T3 (10.39%), and 174 students at T4 (12.65%). When valid scores on both focal scales were required, the available sample sizes were 1,374 at T1, 1,291 at T2, 1,227 at T3, and 1,190 at T4.

Response-pattern analyses showed that 1,073 students completed all four waves, accounting for 77.98% of the baseline sample. The most common incomplete patterns were missing only T4 (*n* = 103, 7.49%), missing T3 but returning at T4 (n = 80, 5.81%), and missing T2 but completing both T3 and T4 (*n* = 47, 3.42%). Other intermittent or sustained missing-data patterns were less frequent, involving 73 students in total (5.31%).

Attrition was not completely random. Compared with students who completed all four waves, students in the incomplete or attrition group differed significantly on baseline sex, highest parental education, CQ1, and GST1. The sex difference was significant, χ^2^(1) = 6.304, *p* = 0.012; the difference in highest parental education was also significant, χ^2^(2) = 7.678, *p* = 0.0215. Baseline CQ1 differed significantly between the complete and incomplete groups, t = −3.439, *p* = 0.0006, as did baseline GST1, t = −2.915, *p* = 0.0037. Baseline residence and sibling configuration did not differ significantly between the two groups.

A further logistic regression analysis, with incomplete participation or attrition coded as 1 and complete four-wave participation coded as 0, produced a similar pattern. Female students were more likely to be in the incomplete or attrition group than male students, OR = 1.43, 95% CI [1.10, 1.85], *p* = 0.0075. Students whose highest parental education was college or above also had a higher likelihood of incomplete participation or attrition, OR = 1.54, 95% CI [1.10, 2.16], *p* = 0.0125. Higher baseline CQ was associated with a greater likelihood of later incomplete participation or attrition, OR = 1.19, *p* = 0.0001, and higher baseline GST was also associated with a greater likelihood of later incomplete participation or attrition, OR = 1.25, *p* = 0.0006.

Taken together, the missing-data pattern had systematic baseline predictors and was therefore more consistent with a missing-at-random framework than with a missing-completely-at-random mechanism. The subsequent models used full-information maximum likelihood to retain all available observations, and a covariate-adjusted sensitivity analysis was conducted to examine whether the focal RI-CLPM estimates were robust to observed demographic differences.

The achieved sample size was appropriate for the planned four-wave bivariate random-intercept cross-lagged panel model (RI-CLPM). The RI-CLPM separates stable between-person differences from within-person fluctuations, which requires adequate sample size and repeated observations for stable estimation of autoregressive and cross-lagged paths (Hamaker et al., 2015). Because statistical power in RI-CLPM depends on several design factors, including the number of waves, sample size, measurement reliability, the proportion of between-person variance, and the expected size of within-person cross-lagged effects, simulation-based planning is recommended for RI-CLPM studies (Mulder, 2023). The present study was designed to recruit more than 1,200 valid baseline participants to maintain adequate statistical precision after expected attrition. The achieved baseline sample of 1,376 students exceeded this target, and 1,202 students still provided at least some item-level data at T4, representing 87.35% of the baseline sample.

### 2.2 Measurements

All measures were administered in Chinese at each wave using the same wording, response format, and scoring procedures. The two focal constructs were other-gender peer contact quality in PE (CQ) and gender-stereotypical trait norms (GST). Wave-specific scores were calculated by averaging the relevant items and labeled CQ1–CQ4 and GST1–GST4. Complete Chinese wording, English reporting translations, response options, scoring rules, and sources are provided in Supplementary Table S1.

Other-gender peer contact quality in PE (CQ) was assessed with the four-item contact-quality component of the Intergroup Contact Experience Scale, based on the Mandarin adaptation used in prior Chinese research (Islam and Hewstone, 1993; Yuan et al., 2024). The referent was classmates of a different gender in PE during the past 4 weeks. The four 7-point semantic-differential pairs were distant–close, competitive–cooperative, unequal–equal, and unpleasant–pleasant. All items were scored from a less positive to a more positive experience. A CQ mean was calculated when at least three of the four items were available; higher scores indicated better perceived contact quality.

Gender-stereotypical trait norms (GST) were measured with the seven-item Gender Stereotypical Traits Scale from the Global Early Adolescent Study, following the item set previously used among adolescents in Shanghai, China (Moreau et al., 2021; Yu et al., 2022). The items assess prescriptive norms concerning boys’ toughness and emotional restraint, girls’ modesty and need for protection, and gender-conforming behavior. Responses ranged from 1 = strongly disagree to 5 = strongly agree. A GST mean was calculated when at least six items were available; no items were reverse scored, and higher scores indicated stronger endorsement of gender-stereotypical trait norms.

### 2.3 Data analysis

Structural equation models were estimated in Mplus Version 8.3 using robust maximum likelihood (MLR) (Muthén and Muthén, 1998–2017). Descriptive statistics, Cronbach’s alpha, model-based McDonald’s omega, Pearson correlations, and repeated-measures intraclass correlations were calculated in R. Model-based omega was estimated from each wave’s one-factor continuous MLR confirmatory factor model (Hayes and Coutts, 2020). CQ required at least three of four items and GST at least six of seven items for a wave-specific score.

Missing observations in the structural models were handled with full-information maximum likelihood, retaining all available data under a missing-at-random assumption (Enders and Bandalos, 2001). Model fit was evaluated jointly using χ^2^, the comparative fit index (CFI), Tucker–Lewis index (TLI), root-mean-square error of approximation (RMSEA) with its 90% confidence interval, and standardized root-mean-square residual (SRMR). CFI and TLI values near 0.95 or higher, RMSEA near 0.06 or lower, and SRMR near 0.08 or lower were treated as approximate guidelines rather than rigid cutoffs (Hu and Bentler, 1999). All tests were two-tailed with *p* < 0.05.

Longitudinal measurement invariance was examined separately for CQ and GST using item-level confirmatory factor analysis. Configural models specified the same factor structure across waves; metric models constrained corresponding loadings to equality; scalar models additionally constrained corresponding intercepts. Residuals of the same item were allowed to correlate across occasions. Invariance was evaluated with changes in CFI, RMSEA, and SRMR following Chen (2007). Item loadings and full model results are reported in Supplementary Tables S2, S3.

Measurement comparability across boys and girls was then examined in a two-group extension of the four-wave CFA. For each construct, the sequence progressed from a common configural form to longitudinal metric and scalar invariance within each sex, followed by equality of the longitudinally constrained loadings and intercepts across sex. Correlated residuals for the same item across waves were retained. Decisions considered global fit, changes in CFI, RMSEA, and SRMR, and MLR-scaled χ^2^ difference tests.

After scalar invariance was established, a conventional cross-lagged panel model and a freely estimated RI-CLPM were fitted. In the RI-CLPM, four repeated scores loaded at 1 on construct-specific random intercepts and on wave-specific within-person factors; observed residual variances were fixed to zero. The random intercepts were allowed to correlate and were orthogonal to the within-person factors. T1 within-person factors and wave-specific disturbances at T2–T4 were allowed to covary. Autoregressive and cross-lagged paths were freely estimated across intervals before temporal constraints were evaluated (Hamaker et al., 2015; Mulder and Hamaker, 2021).

Temporal stationarity was evaluated through a hierarchy beginning with the freely estimated RI-CLPM. Autoregressive paths were constrained by construct, equality of CQ-to-GST paths was then added, and equality of GST-to-CQ paths was added last. A model constraining only the two cross-lagged families was also estimated. Because MLR was used, nested comparisons employed Satorra–Bentler scaled χ^2^ difference tests; path-family equality and contrasts involving the summer-spanning T2–T3 interval were also evaluated with Wald tests. Unstandardized coefficients were constrained because standardized estimates depend on wave-specific variances. AIC and BIC were reported descriptively as comparative parsimony indices (Akaike, 1974; Schwarz, 1978).

The two cross-lagged directions were compared directly after CQ and GST were placed on common four-wave scales using one pooled mean and standard deviation for each construct. The CQ-to-GST minus GST-to-CQ difference was tested at each interval and averaged across intervals; a joint three-degree-of-freedom Wald test assessed equality of the corresponding directions. Holm adjustment was applied to the three interval-specific comparisons. These tests concern asymmetry in fitted prospective associations and do not identify causal asymmetry.

The sensitivity-analysis set included a covariate-adjusted RI-CLPM, 44-indicator and 40-indicator multiple-indicator models, an equality-item-excluded score model, exhaustive leave-one-indicator-out models, one-factor and correlated two-factor measurement models, complete-case and inverse-probability-weighted models, and an attrition-pattern model. In the covariate-adjusted model, sex, residence, sibling configuration, and highest parental education predicted both random intercepts and the T1 within-person factors. Detailed specifications and results are provided in Supplementary Tables S4–S12.

After cross-sex scalar invariance was supported, a score-based multigroup RI-CLPM compared boys and girls. A free model allowed all autoregressive and cross-lagged paths to vary across time and sex; within-sex temporal equality and cross-sex equality of path families were then evaluated with MLR-scaled difference tests and Wald tests applied to unstandardized coefficients. Standardized coefficients were used for descriptive reporting, and Holm adjustment was applied to interval-specific sex contrasts. A 44-indicator multigroup RI-CLPM provided an item-level sensitivity check. Sex-group measurement and structural results are reported in Supplementary Tables S13–S15.

## 3 Results

### 3.1 Preliminary analyses, descriptive statistics, and reliability

Table 2 presents descriptive statistics, reliability estimates, repeated-measures intraclass correlations, and zero-order correlations. Scale-specific sample sizes declined from 1,376 for CQ and 1,374 for GST at T1 to 1,201 and 1,191, respectively, at T4. CQ means ranged from 4.308 to 4.413, and GST means ranged from 3.359 to 3.388.

**Table 2:** Descriptive statistics, reliability estimates, intraclass correlations, and zero-order correlations.

No.	Variable	*n*	M	SD	α	ω	ICC	1	2	3	4	5	6	7	8
1	CQ (T1)	1,376	4.407	1.581	0.904	0.904	0.449	—							
2	CQ (T2)	1,295	4.413	1.592	0.901	0.901	0.449	0.506	—						
3	CQ (T3)	1,232	4.308	1.575	0.901	0.901	0.449	0.430	0.527	—					
4	CQ (T4)	1,201	4.361	1.585	0.906	0.907	0.449	0.351	0.392	0.506	—				
5	GST (T1)	1,374	3.388	1.064	0.932	0.932	0.487	−0.181	−0.114	−0.073	−0.062	—			
6	GST (T2)	1,291	3.359	1.068	0.932	0.932	0.487	−0.258	−0.195	−0.116	−0.080	0.546	—		
7	GST (T3)	1,228	3.366	1.087	0.936	0.936	0.487	−0.236	−0.246	−0.192	−0.113	0.417	0.546	—	
8	GST (T4)	1,191	3.360	1.067	0.935	0.935	0.487	−0.144	−0.186	−0.217	−0.160	0.386	0.482	0.542	—

CQ, other-gender peer contact quality; GST, gender-stereotypical trait norms; T1–T4, Waves 1–4; M, mean; SD, standard deviation; α, Cronbach’s alpha; ω, model-based McDonald’s omega from a one-factor continuous MLR CFA; ICC, repeated-measures intraclass correlation coefficient. Correlations are shown below the diagonal. Sample sizes vary because of wave- and scale-specific missing responses. The same construct-level ICC is shown across waves.

Internal consistency was strong across waves. Cronbach’s *α* ranged from 0.901 to 0.906 for CQ and from 0.932 to 0.936 for GST. Model-based McDonald’s *ω* was 0.904, 0.901, 0.901, and 0.907 for CQ and 0.932, 0.932, 0.936, and 0.935 for GST at T1–T4. The repeated-measures intraclass correlations were 0.449 for CQ and 0.487 for GST, indicating substantial stable between-person variance alongside meaningful within-person variation.

Within-construct correlations were positive and moderate across waves, ranging from 0.351 to 0.527 for CQ and from 0.386 to 0.546 for GST. Concurrent CQ–GST correlations were negative at each wave (−0.160 to −0.195), and all cross-construct correlations were negative (−0.062 to −0.258).

### 3.2 Longitudinal measurement invariance

Longitudinal measurement invariance was examined before structural modeling. Configural, metric, and scalar models fit well for both constructs, and changes in CFI, RMSEA, and SRMR remained within the prespecified criteria (Table 3; Supplementary Table S3). All item-level standardized loadings were statistically significant and are reported in Supplementary Table S2.

**Table 3:** Longitudinal measurement invariance of CQ and GST across four waves.

Variable	Model	χ^2^	df	*p*	CFI	TLI	RMSEA	RMSEA 90% CI	SRMR	ΔCFI	ΔRMSEA	ΔSRMR
CQ	Configural	59.061	74	0.897	1.000	1.002	0.000	[0.000, 0.007]	0.012	—	—	—
	Metric	65.369	83	0.923	1.000	1.002	0.000	[0.000, 0.005]	0.014	0.000	0.000	0.002
	Scalar	78.551	92	0.840	1.000	1.001	0.000	[0.000, 0.009]	0.015	0.000	0.000	0.001
GST	Configural	375.018	302	0.003	0.997	0.996	0.013	[0.008, 0.017]	0.016	—	—	—
	Metric	397.297	320	0.002	0.997	0.996	0.013	[0.008, 0.017]	0.018	0.000	0.000	0.002
	Scalar	407.960	338	0.005	0.997	0.997	0.012	[0.007, 0.016]	0.019	0.000	−0.001	0.001

CQ, other-gender peer contact quality; GST, gender-stereotypical trait norms; CFI, comparative fit index; TLI, Tucker–Lewis index; RMSEA, root-mean-square error of approximation; CI, confidence interval; SRMR, standardized root-mean-square residual. Change indices compare each model with the immediately preceding, less restrictive model within the same construct.

The CQ scalar model yielded χ^2^(92) = 78.551, *p* = 0.840, CFI = 1.000, TLI = 1.001, RMSEA = 0.000, 90% CI [0.000, 0.009], and SRMR = 0.015. The GST scalar model yielded χ^2^(338) = 407.960, *p* = 0.005, CFI = 0.997, TLI = 0.997, RMSEA = 0.012, 90% CI [0.007, 0.016], and SRMR = 0.019. Full scalar invariance was therefore retained for both measures.

Measurement comparability across boys and girls was also supported. For CQ, the cross-sex scalar model fit exceptionally well, χ^2^(190) = 184.040, *p* = 0.608, CFI = 1.000, TLI = 1.001, RMSEA = 0.000, 90% CI [0.000, 0.015], and SRMR = 0.023. Equality of loadings, scaled Δχ^2^(3) = 2.491, *p* = 0.477, and intercepts, scaled Δχ^2^(3) = 1.627, *p* = 0.653, was supported. For GST, the scalar model fit well, χ^2^(688) = 764.358, *p* = 0.023, CFI = 0.997, TLI = 0.997, RMSEA = 0.013, 90% CI [0.005, 0.018], and SRMR = 0.027; equality of loadings, scaled Δχ^2^(6) = 5.469, *p* = 0.485, and intercepts, scaled Δχ^2^(6) = 4.477, *p* = 0.612, was likewise supported. Full scalar-invariance results are reported in Supplementary Table S13.

### 3.3 Model comparison

Model comparison proceeded in two stages. The conventional cross-lagged panel model was compared descriptively with the freely estimated RI-CLPM to assess the importance of separating stable between-person differences. Temporal stationarity within the RI-CLPM was then evaluated with formal scaled difference tests and path-family Wald tests. Freely estimated lagged coefficients are reported in Supplementary Table S4, and the full stationarity sequence is reported in Supplementary Table S5.

The conventional model provided comparatively poor fit, χ^2^(12) = 251.403, *p* < 0.001, CFI = 0.909, TLI = 0.795, RMSEA = 0.120, 90% CI [0.108, 0.134], and SRMR = 0.064. The freely estimated RI-CLPM fit well, χ^2^(9) = 27.689, *p* = 0.001, CFI = 0.993, TLI = 0.978, RMSEA = 0.039, 90% CI [0.023, 0.056], and SRMR = 0.022 (Table 4).

**Table 4:** Model comparison and fit indices for the conventional CLPM and RI-CLPMs.

Model	Constraint	χ^2^ (df)	*p*	CFI	TLI	SRMR	RMSEA [90% CI]	ΔCFI	ΔRMSEA	ΔSRMR	AIC	BIC
M0	Conventional CLPM with freely estimated lagged paths	251.403 (12)	< 0.001	0.909	0.795	0.064	0.120 [0.108, 0.134]	—	—	—	31,796.319	31,963.581
M1	RI-CLPM with freely estimated lagged paths	27.689 (9)	0.001	0.993	0.978	0.022	0.039 [0.023, 0.056]	—	—	—	31,566.922	31,749.865
M2	RI-CLPM with autoregressive and cross-lagged paths constrained equal	29.258 (17)	0.032	0.995	0.992	0.023	0.023 [0.007, 0.037]	0.002	−0.016	0.001	31,552.750	31,693.877

CLPM, cross-lagged panel model; RI-CLPM, random-intercept cross-lagged panel model; CFI, comparative fit index; TLI, Tucker–Lewis index; SRMR, standardized root-mean-square residual; RMSEA, root-mean-square error of approximation; CI, confidence interval; AIC, Akaike information criterion; BIC, Bayesian information criterion. The formal MLR-scaled comparison of M2 with M1 was Δχ^2^(8) = 1.869, *p* = 0.985. Lower AIC and BIC indicate comparatively better fit after accounting for model complexity.

Constraining all autoregressive and cross-lagged path families to equality did not worsen fit relative to the free RI-CLPM, scaled Δχ^2^(8) = 1.869, *p* = 0.985. Separate Wald tests were nonsignificant for CQ autoregression (*p* = 0.994), GST autoregression (*p* = 0.775), CQ-to-GST paths (*p* = 0.535), and GST-to-CQ paths (*p* = 0.872). None of the eight contrasts comparing the summer-spanning T2–T3 path with adjacent intervals was significant. The equal-lag RI-CLPM retained excellent fit, χ^2^(17) = 29.258, *p* = 0.032, CFI = 0.995, TLI = 0.992, RMSEA = 0.023, and SRMR = 0.023, and was retained as a parsimonious summary.

### 3.4 Between-person and within-person associations in the final RI-CLPM

**Figure 2:**
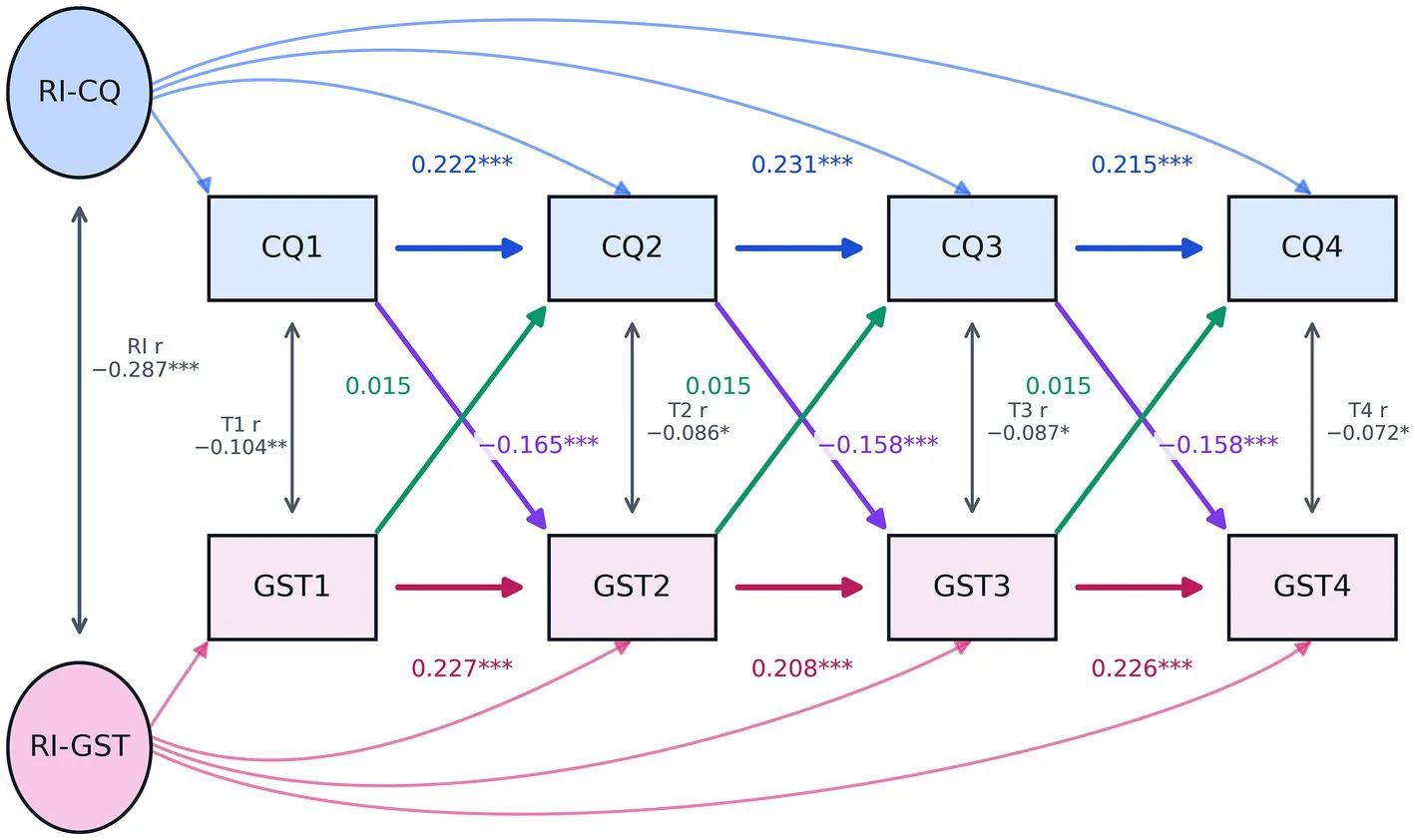
Standardized estimates from the final equal-lag RI-CLPM. Values are STDYX standardized estimates. CQ, other-gender peer contact quality; GST, gender-stereotypical trait norms; RI, random intercept; T1–T4, Waves 1–4. RI *r* denotes the between-person correlation between the CQ and GST random intercepts; T1 *r*–T4 *r* denote within-wave CQ–GST correlations among within-person factors. Blue paths indicate CQ autoregressive effects, pink paths indicate GST autoregressive effects, purple paths indicate CQ-to-GST cross-lagged effects, and green paths indicate GST-to-CQ cross-lagged effects. Equality constraints were imposed on the unstandardized autoregressive and cross-lagged coefficients; therefore, the corresponding standardized estimates may vary slightly across intervals. * *p* < 0.05, ** *p* < 0.01, *** *p* < 0.001.

**Figure 3:**
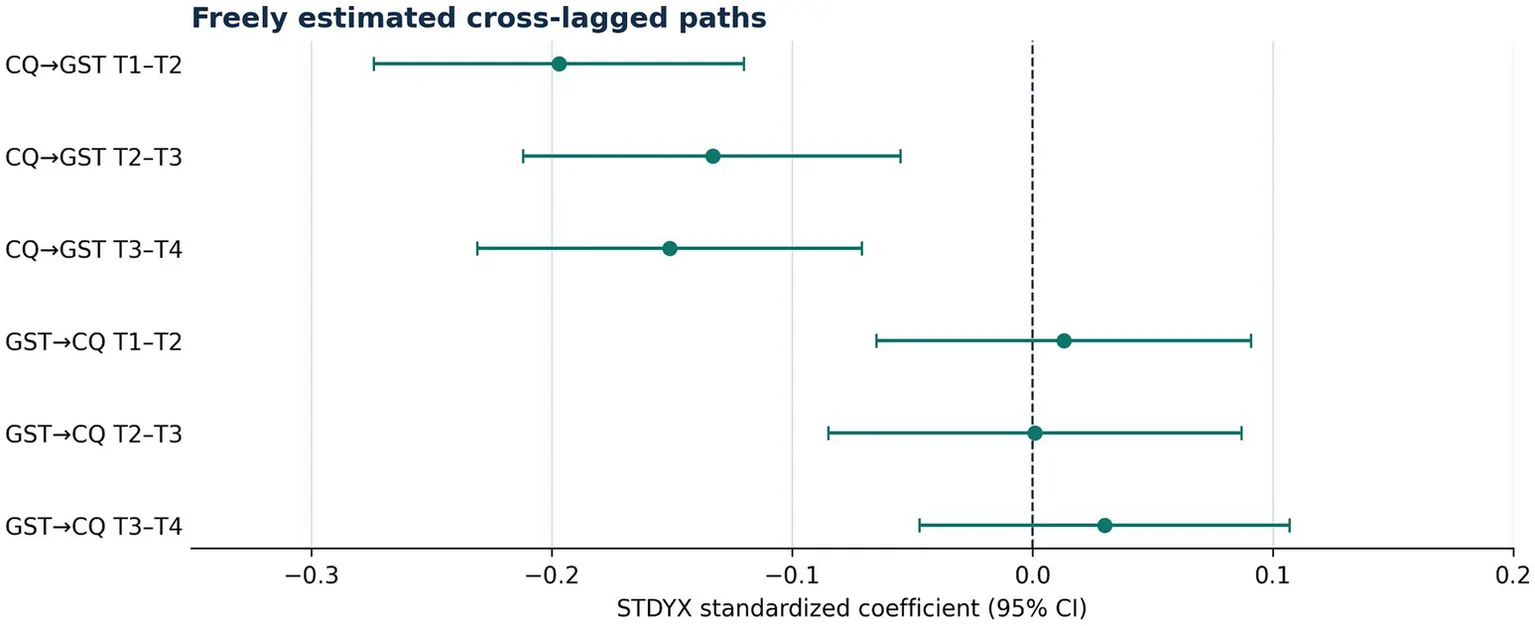
Forest plot of freely estimated standardized cross-lagged effects. Points represent STDYX standardized estimates from the unconstrained RI-CLPM, and horizontal lines represent 95% confidence intervals. The dashed vertical line indicates zero. CQ, other-gender peer contact quality; GST, gender-stereotypical trait norms; T1–T4, Waves 1–4.

Table 5 and Figure 2 present the equal-lag RI-CLPM as a parsimonious summary. The unconstrained empirical pattern is shown by the freely estimated coefficients in Supplementary Table S4 and Figure 3.

**Table 5:** Unstandardized and standardized estimates from the final RI-CLPM with equal lagged paths.

Path	Interval	b	SE	*β*/r	Standardized SE	95% CI for *β*/r	*p*
RI-CQ ↔ RI-GST	Between-person	−0.185	0.037	−0.287	0.050	[−0.385, −0.189]	< 0.001
W-CQ1 → W-CQ2	T1–T2	0.224	0.032	0.222	0.031	[0.161, 0.283]	< 0.001
W-CQ2 → W-CQ3	T2–T3	0.224	0.032	0.231	0.033	[0.166, 0.296]	< 0.001
W-CQ3 → W-CQ4	T3–T4	0.224	0.032	0.215	0.033	[0.150, 0.280]	< 0.001
W-GST1 → W-GST2	T1–T2	0.219	0.034	0.227	0.033	[0.162, 0.292]	< 0.001
W-GST2 → W-GST3	T2–T3	0.219	0.034	0.208	0.033	[0.143, 0.273]	< 0.001
W-GST3 → W-GST4	T3–T4	0.219	0.034	0.226	0.035	[0.157, 0.295]	< 0.001
W-CQ1 → W-GST2	T1–T2	−0.105	0.017	−0.165	0.027	[−0.218, −0.112]	< 0.001
W-CQ2 → W-GST3	T2–T3	−0.105	0.017	−0.158	0.026	[−0.209, −0.107]	< 0.001
W-CQ3 → W-GST4	T3–T4	−0.105	0.017	−0.158	0.026	[−0.209, −0.107]	< 0.001
W-GST1 → W-CQ2	T1–T2	0.022	0.039	0.015	0.026	[−0.036, 0.066]	0.567
W-GST2 → W-CQ3	T2–T3	0.022	0.039	0.015	0.025	[−0.034, 0.064]	0.565
W-GST3 → W-CQ4	T3–T4	0.022	0.039	0.015	0.026	[−0.036, 0.066]	0.566

RI-CLPM = random-intercept cross-lagged panel model; CQ, other-gender peer contact quality; GST, gender-stereotypical trait norms; RI, random intercept; W, within-person latent deviation; T1–T4, Waves 1–4; CI, confidence interval. For directional paths, b denotes the unstandardized regression coefficient and *β* denotes the STDYX standardized coefficient. For the association between RI-CQ and RI-GST, b is the unstandardized covariance and r is the standardized correlation. Unstandardized autoregressive and cross-lagged coefficients were constrained to equality across the three intervals. Standardized coefficients differ slightly across intervals because wave-specific variances were not identical.

At the stable between-person level, the CQ and GST random intercepts were negatively correlated, r = −0.287, 95% CI [−0.385, −0.189], *p* < 0.001. Students who generally experienced higher-quality interactions with other-gender peers therefore tended to report lower overall endorsement of GST across the study period.

Within-person autoregressive paths were positive for both constructs. In the equal-lag summary model, standardized CQ autoregressive estimates were *β* = 0.222, 0.231, and 0.215, and GST estimates were *β* = 0.227, 0.208, and 0.226 across the three transitions, respectively (all *p* < 0.001).

In the freely estimated RI-CLPM, higher-than-usual CQ was associated with lower subsequent GST at every interval: T1–T2, b = −0.127, SE = 0.026, 95% CI [−0.178, −0.077], *β* = −0.197, *p* < 0.001; T2–T3, b = −0.090, SE = 0.027, 95% CI [−0.143, −0.037], β = −0.133, *p* = 0.001; and T3–T4, b = −0.101, SE = 0.027, 95% CI [−0.155, −0.047], *β* = −0.151, *p* < 0.001. All unstandardized and standardized confidence intervals excluded zero.

The freely estimated reverse GST-to-CQ paths were small and nonsignificant: β = 0.013, 95% CI [−0.065, 0.091], *p* = 0.746; β = 0.001, 95% CI [−0.085, 0.087], *p* = 0.986; and β = 0.030, 95% CI [−0.047, 0.107], *p* = 0.444 for T1–T2, T2–T3, and T3–T4, respectively.

Direct comparisons on the common scales supported a directional difference. CQ-to-GST minus GST-to-CQ differences were −0.201 (SE = 0.051, 95% CI [−0.300, −0.102], Holm-adjusted *p* < 0.001), −0.134 (SE = 0.055, 95% CI [−0.241, −0.027], adjusted *p* = 0.014), and −0.179 (SE = 0.055, 95% CI [−0.287, −0.072], adjusted *p* = 0.002). The average difference was −0.171, SE = 0.026, 95% CI [−0.222, −0.121], *p* < 0.001, and the joint test was Wald χ^2^(3) = 44.574, *p* < 0.001 (Supplementary Table S6). These results indicate temporal asymmetry in the fitted prospective associations, without establishing causal asymmetry.

### 3.5 Sensitivity analysis with demographic covariates

A covariate-adjusted sensitivity model added sex, residence, sibling configuration, and highest parental education as time-invariant predictors of the CQ and GST random intercepts and the T1 within-person factors. Model fit and adjusted cross-lagged estimates are shown in Table 6 and Supplementary Table S12.

**Table 6:** Covariate sensitivity analysis: Model fit and adjusted cross-lagged estimates.

Panel A. Model fit.									
Model	*n*	χ^2^ (df)	*p*	CFI	TLI	SRMR	RMSEA [90% CI]	AIC	BIC
Final RI-CLPM equal lags	1,376	29.258 (17)	0.032	0.995	0.992	0.023	0.023 [0.007, 0.037]	31,552.750	31,693.877
Covariate sensitivity model	1,376	51.483 (41)	0.126	0.996	0.993	0.019	0.014 [0.000, 0.024]	31,586.496	31,853.070

Panel B. Adjusted cross-lagged estimates						
Path	Interval	Final *β*	Adjusted *β*	95% CI for adjusted *β*	*p*	Δ*β*
CQ → GST	T1–T2	−0.165	−0.166	[−0.219, −0.113]	< 0.001	−0.001
CQ → GST	T2–T3	−0.158	−0.158	[−0.209, −0.107]	< 0.001	0.000
CQ → GST	T3–T4	−0.158	−0.159	[−0.210, −0.108]	< 0.001	−0.001
GST → CQ	T1–T2	0.015	0.014	[−0.037, 0.065]	0.588	−0.001
GST → CQ	T2–T3	0.015	0.014	[−0.035, 0.063]	0.586	−0.001
GST → CQ	T3–T4	0.015	0.014	[−0.037, 0.065]	0.587	−0.001

CQ, other-gender peer contact quality; GST, gender-stereotypical trait norms; RI-CLPM, random-intercept cross-lagged panel model; CFI, comparative fit index; TLI, Tucker–Lewis index; SRMR, standardized root-mean-square residual; RMSEA, root-mean-square error of approximation; CI, confidence interval; AIC, Akaike information criterion; BIC, Bayesian information criterion. The covariate sensitivity model adjusted for sex, residence, sibling configuration, and highest parental education as time-invariant covariates predicting the random intercepts and T1 within-person factors. Δβ, adjusted β minus final-model β. AIC and BIC are reported descriptively because the covariate sensitivity model includes additional observed predictors and was estimated to evaluate robustness rather than to replace the final RI-CLPM.

The adjusted model fit well, χ^2^(41) = 51.483, *p* = 0.126, CFI = 0.996, TLI = 0.993, RMSEA = 0.014, 90% CI [0.000, 0.024], and SRMR = 0.019. CQ-to-GST estimates were *β* = −0.166, −0.158, and −0.159 (all *p* < 0.001), whereas reverse estimates were *β* = 0.014 at each transition (*p* = 0.586–0.588). The largest absolute change from the unadjusted equal-lag estimates was |Δβ| = 0.001. The estimates therefore changed little after adjustment for the four measured demographic covariates.

Girls showed a small lower stable CQ level than boys, *β* = −0.075, *p* = 0.049; no other demographic coefficient predicting the random intercepts or T1 within-person factors was significant. This isolated, borderline association was treated as exploratory and does not establish moderation of the structural paths.

### 3.6 Measurement, item-overlap, and attrition sensitivity analyses

The 44-indicator multiple-indicator RI-CLPM directly represented item-level measurement error. The freely estimated model fit well, χ^2^(871) = 996.718, CFI = 0.997, TLI = 0.997, RMSEA = 0.010, and SRMR = 0.023. Its CQ-to-GST paths were *β* = −0.232, −0.170, and −0.179 (all *p* < 0.001), whereas reverse paths were *β* = 0.015, 0.001, and 0.034 (all nonsignificant). Equality constraints remained compatible with the data, scaled Δχ^2^(8) = 1.829, *p* = 0.986 (Supplementary Table S7).

The CQ equality indicator was the third item, unequal–equal. After it was removed at all four waves, the score-based CQ-to-GST paths remained negative and significant: *β* = −0.199, −0.140, and −0.127 (*p* ≤ 0.002); reverse paths were *β* = 0.007, −0.011, and 0.032 (all nonsignificant). Temporal equality remained compatible with the data, scaled Δχ^2^(8) = 3.166, *p* = 0.924. The corresponding 40-indicator multiple-indicator models also fit well, and their equality test was nonsignificant, scaled Δχ^2^(8) = 3.501, *p* = 0.899 (Supplementary Table S8).

The longitudinal correlated two-factor model strongly outperformed a one-factor model, scaled Δχ^2^(28) = 8654.008, *p* < 0.001; the two-factor model fit well, χ^2^(862) = 973.120, CFI = 0.997, TLI = 0.997, RMSEA = 0.010, and SRMR = 0.019, supporting construct distinctiveness. Exhaustive leave-one-indicator-out analyses also retained negative CQ-to-GST and nonsignificant reverse paths across all four deletion scenarios (Supplementary Tables S9, S10).

Attrition analyses produced the same substantive pattern. Among 1,054 students with all eight scale scores, the equal CQ-to-GST coefficient was b = −0.096; raw inverse-probability weighting, truncated weighting, and an attrition-pattern model yielded b = −0.093, −0.094, and −0.105, respectively (all *p* < 0.001). Reverse paths remained nonsignificant. These analyses address observed completion predictors but cannot rule out dropout related to unobserved values (Supplementary Table S11).

### 3.7 Sex-group measurement and structural analyses

The freely estimated two-group score-based RI-CLPM included 712 boys and 664 girls and fit acceptably, χ^2^(18) = 34.020, *p* = 0.013, CFI = 0.994, TLI = 0.981, RMSEA = 0.036, 90% CI [0.016, 0.054], and SRMR = 0.024. Standardized CQ-to-GST paths were *β* = −0.162, −0.142, and −0.138 for boys and *β* = −0.220, −0.119, and −0.179 for girls across T1–T2, T2–T3, and T3–T4. The joint sex-group test was nonsignificant, Wald χ^2^(3) = 1.168, *p* = 0.761. The corresponding GST-to-CQ paths were *β* = 0.071, 0.008, and −0.005 for boys and *β* = −0.042, −0.004, and 0.056 for girls; their joint sex-group test was also nonsignificant, Wald χ^2^(3) = 2.704, *p* = 0.440. None of the interval-specific contrasts was significant after Holm adjustment (Supplementary Table S14).

Temporal equality within sex was compatible with the data, scaled Δχ^2^(16) = 10.190, *p* = 0.857. Starting from the stationary model, cross-sex equality constraints were supported for CQ autoregression (*p* = 1.000), GST autoregression (*p* = 0.123), CQ-to-GST paths (*p* = 0.533), GST-to-CQ paths (*p* = 0.827), both cross-lagged families jointly (*p* = 0.886), and all lagged paths jointly (*p* = 0.719). The stable CQ–GST correlations were r = −0.290 in both groups, and the random-intercept covariance did not differ detectably, Wald χ^2^(1) = 0.012, *p* = 0.912.

The 44-indicator multigroup RI-CLPM also fit well, χ^2^(1760) = 1976.425, *p* < 0.001, CFI = 0.995, TLI = 0.994, RMSEA = 0.013, and SRMR = 0.031. Cross-sex equality was supported for CQ-to-GST paths, scaled Δχ^2^(3) = 1.225, *p* = 0.747; GST-to-CQ paths, scaled Δχ^2^(3) = 2.416, *p* = 0.491; and both directions jointly, scaled Δχ^2^(6) = 4.188, *p* = 0.651 (Supplementary Table S15). The analyses therefore found no statistically detectable sex-group moderation of the focal paths; this result supports the pooled model as a parsimonious sample-level summary but does not demonstrate exact equivalence.

## 4 Discussion

This study separated stable between-person covariation from within-person prospective associations between CQ and GST in coeducational PE. Students with generally higher CQ tended to endorse lower GST, and higher-than-usual CQ was followed by lower-than-usual GST at the next wave. The reverse paths were close to zero. Formal comparisons supported temporal equality of the lagged paths and showed that the CQ-to-GST direction was more negative than the corresponding reverse direction. The pattern remained when measurement error was modeled, the equality item was removed, observed demographics were included, and alternative attrition treatments were applied. Full cross-sex measurement invariance and multigroup structural tests found no statistically detectable differences in the focal paths between boys and girls.

Because individual age, grade, and school-stage information was unavailable, the estimates characterize the pooled sample. Whether the CQ–GST association was present or comparable across primary- and secondary-school stages remains unknown, and developmental invariance across childhood and adolescence cannot be inferred.

### 4.1 What the study clarifies about contact quality and gender norms

The findings clarify the distinction between mixed-gender co-presence and the quality of interaction. Coeducational placement alone does not indicate whether students experience trust, cooperation, equal participation, or respect. The negative between-person and prospective within-person associations centered on these relational qualities, consistent with a quality-focused interpretation of gendered contact rather than an exposure-only interpretation. This conclusion is especially relevant to PE, where performance, role allocation, dependence on teammates, and reactions to mistakes are publicly visible.

The results also clarify the level at which the association appears. The random-intercept correlation describes stable differences among students and may reflect enduring relational environments, family socialization, school climate, or dispositions. The cross-lagged paths describe a separate pattern: temporary deviations in a student’s CQ contained prospective information about that student’s later GST after stable differences were separated. The RI-CLPM therefore strengthens level-specific interpretation, although it does not convert the association into a causal effect.

The freely estimated CQ-to-GST coefficients varied in magnitude but were negative at all three transitions, and formal scaled and Wald tests found no evidence that equality constraints were incompatible with the data. The summer-spanning T2–T3 paths also did not differ detectably from adjacent transitions. Temporal equality therefore provides a parsimonious summary of the observed intervals. Independent replication at each transition and constancy beyond the sampled lags remain unestablished.

The measurement results support the stability and distinctiveness of the focal association in two respects. The multiple-indicator RI-CLPM preserved the direction of the score-based estimates when item-level measurement error was represented. The CQ-to-GST paths also remained negative and significant after the unequal–equal indicator was removed, although the T3–T4 coefficient was somewhat smaller. This pattern indicates that the association was not attributable to the equality item alone, while equality may contribute to its magnitude at some intervals. The correlated two-factor model further supported the empirical distinction between CQ and GST.

The sex-group analyses add a separate form of measurement and structural evidence. Full metric and scalar invariance supported comparison of boys and girls, and formal tests did not detect differences in either cross-lagged direction. The pooled coefficients are therefore defensible as parsimonious averages for the sampled boys and girls, although nonsignificant difference tests do not prove that the underlying effects are exactly equivalent.

The temporal ordering in the RI-CLPM is compatible with several processes proposed by contact theory and gender-schema accounts, including reduced gender-related anxiety, greater perspective taking, exposure to counter-stereotypical competence, increased interpersonal trust, teacher norm signaling, and changes in role expectations. Direct measures of these processes were unavailable, leaving their relative contribution and mediating role unresolved.

### 4.2 Interpreting the directional asymmetry

The reverse GST-to-CQ coefficients were small and imprecise at the examined lags. Nonsignificance alone would not demonstrate asymmetry, but the direct common-scale comparisons showed that the CQ-to-GST paths were more negative than their corresponding reverse paths. The supported conclusion is therefore a temporally asymmetric prospective association, not proof that GST has no behavioral consequences.

One potential account of the directional pattern concerns the institutional organization of PE. Teacher-assigned groups and activities may limit students’ ability to select or avoid contact, which could weaken an attitude-based selection pathway. Broad GST may still be expressed through dominance, role allocation, trust, avoidance, or other lesson-level behavior without producing detectable changes in a global four-item evaluation of contact quality several weeks later. The contribution of teacher behavior, grouping practices, and students’ control over interaction remains unresolved because these features were not measured.

Timing and specificity remain important boundary conditions. Norms may affect interaction within a single lesson or shape friendship networks over longer periods, while the present waves represented several weeks of school-based PE. The absence of a reverse path at these intervals cannot be generalized to all temporal scales or to more behaviorally specific outcomes.

The directional tests also do not establish causal ordering. A curriculum change, teacher intervention, activity type, group reorganization, peer conflict, injury, or changing classroom climate could affect CQ and GST during the same interval. The direct contrasts show a difference between fitted prospective coefficients under the specified model; they do not eliminate time-varying confounding or reciprocal environmental influence.

Demographic and attrition analyses narrow but do not remove this uncertainty. Adjustment for sex, residence, sibling configuration, and parental education changed the focal estimates very little, and complete-case and weighted analyses preserved the pattern. These checks do not represent family gender socialization, school resources, teacher attitudes, classroom climate, or unobserved causes of dropout.

The fitted paths are compatible with a context-dependent account in which reciprocity may vary according to how contact is organized. The present data support a difference between the two prospective coefficients within the sampled setting, but they do not establish why that difference arose. Reciprocity should therefore remain an empirical question whose form may depend on students’ opportunities to select, avoid, and organize interaction.

### 4.3 Implications for multilevel inclusive PE and future intervention research

Within a multilevel PE ecology, CQ represents an interpersonal manifestation of broader participation conditions. Inclusive participation and belonging may reflect peer cooperation and respect, teacher grouping and feedback, pedagogical organization, activity content, access to space and equipment, school norms, and implementation support. Reviews of physical-activity interventions in children and adolescents show that individual and interpersonal determinants have received substantially more attention than organizational and environmental determinants, while effects on targeted determinants and behaviors have generally been limited or uncertain (Ciaccioni et al., 2026; Khudair et al., 2025; Kolovelonis et al., 2024; Ling et al., 2024). Their broader implication is that peer-contact quality is best considered together with teacher, organizational, and environmental conditions.

Future intervention research could evaluate multicomponent packages that combine structured positive interdependence, equitable participation and decision-making, rotation of high-status roles, and explicit responses to ridicule or gender policing with teacher preparation, grouping procedures, activity design, and organizational support. Cooperative-learning reviews provide supporting context for structured interdependence, shared participation, and role organization in PE (Bores-García et al., 2021; Casey and Goodyear, 2015; Zach et al., 2023). Their effects on CQ and GST require direct experimental evaluation.

Cluster-randomized or stepped-wedge trials could compare a prespecified multilevel package with usual PE provision. Repeated measurement should include CQ, GST, observed participation and role allocation, teacher behavior, pedagogical organization, environmental access, implementation fidelity, and potential unintended effects. Such designs could test whether changes at teacher, organizational, or environmental levels alter interpersonal CQ and whether any subsequent change in GST is mediated by CQ. Future observational research should also retain age, grade, school stage, and verified school, class, and PE-teacher identifiers to permit developmental comparisons and multilevel models of shared context.

## 5 Limitations and future directions

The observational design is the central limitation. RI-CLPM separates stable between-person differences from within-person deviations but does not identify causal effects. Unmeasured time-varying factors—including activity content, teacher behavior, group composition, injuries, peer conflict, school events, and classroom climate—could influence both constructs. Directional contrasts and sensitivity analyses do not remove this source of uncertainty (Lüdtke and Robitzsch, 2022; Mulder et al., 2024). These potential confounders operate across interpersonal, teacher, organizational, and environmental levels that were not jointly measured.

Individual age, grade, pubertal or developmental stage, and student-level school-stage information were unavailable and could not be reconstructed from the retained study records. The estimated associations therefore represent pooled averages across students recruited from primary and secondary schools. The study cannot determine whether CQ, GST, or their prospective association differed according to cognitive maturity, peer-network organization, pubertal development, stage-specific gender-norm formation, or PE practices. Age-specific evidence reviews also underscore the value of developmentally and contextually resolved analyses (Kolovelonis et al., 2024; Ling et al., 2024). Verified school, class, and PE-teacher identifiers were likewise unavailable, precluding cluster-robust standard errors and multilevel RI-CLPMs; standard errors may therefore be affected by unmodeled dependence within shared contexts.

The selected lag represents only one timescale. Although formal tests supported equality across the observed transitions and the summer-spanning interval did not differ detectably from adjacent intervals, these findings do not imply a constant process in continuous time. Contact and norm enactment may operate within lessons, across teaching units, or over longer developmental periods.

Both constructs were measured by student self-report, creating potential shared-method and social-desirability bias (Podsakoff et al., 2003). CQ represented four broad qualities and did not separately assess negative contact or observed role allocation. GST captured broad trait norms rather than PE-specific beliefs. The study therefore cannot identify whether associations concerned athletic competence, leadership, protection, emotional expression, or another content domain. Future work should distinguish trait, activity, and role content (Liben and Bigler, 2002).

Reduced gender-related anxiety, perspective taking, counter-stereotypical learning, interpersonal trust, perceived typicality, teacher norm signaling, and changes in role expectations remain candidate explanations. Direct tests require designs that measure their temporal position or manipulate the relevant contact conditions.

The sample came from 20 schools in Chengdu and was not nationally representative. Curricula, resources, teacher preparation, organizational arrangements, and gender norms may differ across regions and educational systems. CQ captured students’ interpersonal perceptions without direct measures of teacher, organizational, or environmental determinants. Sex was recorded in binary categories. Full metric and scalar invariance supported score comparability across boys and girls, and no statistically detectable sex-group differences emerged in the focal paths. Exact equivalence remains uncertain, and the binary classification does not represent gender identities outside that framework.

Attrition had observed baseline predictors. Complete-case, inverse-probability-weighted, and attrition-pattern analyses preserved the focal association, but such methods cannot guarantee unbiased estimates when dropout depends on unobserved values (Little and Rubin, 2019).

## 6 Conclusion

Across four waves, better other-gender peer contact quality in coeducational PE was associated with lower endorsement of gender-stereotypical trait norms at stable between-person and time-specific within-person levels. Freely estimated CQ-to-GST paths were negative across all observed intervals and more negative than the reverse paths. Full measurement invariance was supported across boys and girls, and multigroup analyses found no statistically detectable sex-group differences in the focal paths. The pattern also remained after direct modeling of measurement error and removal of the CQ equality item. These findings describe prospective associations in the pooled sample; they do not identify causal effects, specific psychological or social mechanisms, or uniform patterns across ages, grades, school stages, and educational contexts. Future multilevel research should jointly measure peer, teacher, pedagogical, organizational, and environmental conditions and experimentally evaluate the proposed intervention targets.

## Data Availability

The original contributions presented in the study are included in the article/Supplementary material, further inquiries can be directed to the corresponding author.
