# Supplementary material for: Longitudinal dynamics between other-gender peer contact quality and gender-stereotypical trait norms in coeducational physical education: a four-wave random-intercept cross-lagged panel study
Source: Front Psychol. 2026 Sep 4;17:1923110. doi: 10.3389/fpsyg.2026.1923110 (PMC13585922; doi:10.3389/fpsyg.2026.1923110)
Supplement: Supplementary file 1 [file Table_1.docx]

**Supplementary Materials**

These materials report complete measurement information and supplementary analyses. All models used robust maximum likelihood unless otherwise noted.

# **Table S1. Complete CQ and GST items, response options, scoring, and sources**

| **Construct** | **Item / dimension** | **Chinese wording** | **English reporting translation** | **Response and scoring** |
| --- | --- | --- | --- | --- |
| CQ | CQ1: Closeness | 疏远—亲近 | Distant–close | 7-point semantic differential; 1 = less positive, 7 = more positive |
| CQ | CQ2: Cooperation | 竞争—合作 | Competitive–cooperative | 7-point semantic differential; 1 = less positive, 7 = more positive |
| CQ | CQ3: Equality | 不平等—平等 | Unequal–equal | 7-point semantic differential; 1 = less positive, 7 = more positive |
| CQ | CQ4: Pleasantness | 不愉快—愉快 | Unpleasant–pleasant | 7-point semantic differential; 1 = less positive, 7 = more positive |
| GST | GST1 | 男孩应该从小培养得坚强，这样才能克服生活中的任何困难。 | Boys should be raised to be strong from an early age so that they can overcome any difficulty in life. | 1 = strongly disagree to 5 = strongly agree |
| GST | GST2 | 女孩应该避免提高嗓门，这样才显得有淑女风范。 | Girls should avoid raising their voices so that they appear ladylike. | 1 = strongly disagree to 5 = strongly agree |
| GST | GST3 | 男孩应该始终保护自己，即使这意味着需要打架。 | Boys should always protect themselves, even if that means fighting. | 1 = strongly disagree to 5 = strongly agree |
| GST | GST4 | 女孩应该保持谦逊。 | Girls should be modest. | 1 = strongly disagree to 5 = strongly agree |
| GST | GST5 | 女孩比男孩更需要父母的保护。 | Girls need more protection from their parents than boys do. | 1 = strongly disagree to 5 = strongly agree |
| GST | GST6 | 举止像女孩的男孩会被认为软弱。 | Boys who behave like girls are considered weak. | 1 = strongly disagree to 5 = strongly agree |
| GST | GST7 | 即使内心感到紧张，男孩也应该表现得坚强。 | Even if they feel nervous inside, boys should appear strong. | 1 = strongly disagree to 5 = strongly agree |

Note. CQ was introduced with a referent to classmates of a different gender in PE during the past four weeks. CQ was averaged when at least three of four items were observed; GST was averaged when at least six of seven items were observed. No GST items were reverse scored. The English GST sentences are reporting translations; the questionnaires were administered in Chinese. CQ was based on Islam and Hewstone (1993) and the Mandarin application reported by Yuan et al. (2024). GST followed the GEAS item set reported by Moreau et al. (2021) and used in Shanghai by Yu et al. (2022).

# **Table S2. Item-level standardized factor loadings across four waves**

| **Construct** | **Indicator** | **T1 loading (SE) [95% CI]** | **T2 loading (SE) [95% CI]** | **T3 loading (SE) [95% CI]** | **T4 loading (SE) [95% CI]** |
| --- | --- | --- | --- | --- | --- |
| CQ | CQ1: distant–close | .850 (.009) [.833, .868] | .843 (.009) [.825, .861] | .830 (.010) [.810, .849] | .850 (.010) [.831, .870] |
| CQ | CQ2: competitive–cooperative | .800 (.010) [.780, .819] | .787 (.010) [.767, .808] | .819 (.010) [.799, .839] | .816 (.010) [.797, .835] |
| CQ | CQ3: unequal–equal | .857 (.009) [.840, .874] | .854 (.009) [.836, .872] | .842 (.010) [.822, .861] | .843 (.010) [.824, .862] |
| CQ | CQ4: unpleasant–pleasant | .845 (.010) [.826, .864] | .849 (.009) [.831, .866] | .839 (.010) [.820, .858] | .854 (.009) [.836, .873] |
| GST | GST1 | .843 (.008) [.828, .858] | .838 (.009) [.821, .855] | .853 (.009) [.836, .870] | .845 (.008) [.828, .861] |
| GST | GST2 | .842 (.009) [.824, .859] | .829 (.009) [.811, .847] | .845 (.009) [.828, .862] | .850 (.009) [.832, .867] |
| GST | GST3 | .831 (.009) [.813, .849] | .835 (.009) [.817, .853] | .837 (.009) [.820, .855] | .850 (.009) [.833, .867] |
| GST | GST4 | .833 (.009) [.815, .850] | .830 (.009) [.813, .847] | .844 (.009) [.826, .861] | .832 (.009) [.814, .849] |
| GST | GST5 | .830 (.010) [.811, .848] | .825 (.010) [.806, .845] | .842 (.009) [.825, .859] | .832 (.010) [.813, .851] |
| GST | GST6 | .775 (.010) [.755, .794] | .771 (.011) [.750, .793] | .779 (.011) [.758, .800] | .768 (.011) [.746, .791] |
| GST | GST7 | .745 (.010) [.725, .766] | .762 (.011) [.740, .783] | .758 (.012) [.735, .781] | .756 (.011) [.734, .778] |

*Note.* Values are STDYX standardized loadings from the longitudinal scalar-invariance models. All loadings were p < .001. Corresponding loadings and intercepts were constrained across waves on the unstandardized scale; standardized values vary because wave-specific variances differ.

# **Table S3. Longitudinal measurement invariance**

| **Construct** | **Model** | **χ²** | **df** | **p** | **CFI** | **TLI** | **RMSEA** | **90% CI** | **SRMR** | **ΔCFI** | **ΔRMSEA** | **ΔSRMR** |
| --- | --- | --- | --- | --- | --- | --- | --- | --- | --- | --- | --- | --- |
| CQ | configural | 59.061 | 74 | = .897 | 1.000 | 1.002 | .000 | [.000, .007] | .012 | — | — | — |
| CQ | metric | 65.369 | 83 | = .923 | 1.000 | 1.002 | .000 | [.000, .005] | .014 | .000 | .000 | .002 |
| CQ | scalar | 78.551 | 92 | = .840 | 1.000 | 1.001 | .000 | [.000, .009] | .015 | .000 | .000 | .001 |
| GST | configural | 375.018 | 302 | = .003 | .997 | .996 | .013 | [.008, .017] | .016 | — | — | — |
| GST | metric | 397.297 | 320 | = .002 | .997 | .996 | .013 | [.008, .017] | .018 | .000 | .000 | .002 |
| GST | scalar | 407.960 | 338 | = .005 | .997 | .997 | .012 | [.007, .016] | .019 | .000 | −.001 | .001 |

*Note.* Residuals of the same indicator were correlated across waves. Equality-excluded CQ measurement-invariance results are reported in Supplementary Table S8.

# **Table S4. Freely estimated RI-CLPM lagged paths**

| **Path** | **Interval** | **b** | **SE** | **95% CI for b** | **p** | **β** | **SE** | **95% CI for β** | **p** |
| --- | --- | --- | --- | --- | --- | --- | --- | --- | --- |
| CQ autoregressive | T1–T2 | .220 | .043 | [.136, .304] | < .001 | .218 | .042 | [.136, .300] | < .001 |
| CQ autoregressive | T2–T3 | .222 | .045 | [.133, .310] | < .001 | .228 | .045 | [.139, .317] | < .001 |
| CQ autoregressive | T3–T4 | .226 | .042 | [.143, .308] | < .001 | .218 | .041 | [.137, .298] | < .001 |
| GST autoregressive | T1–T2 | .211 | .040 | [.132, .291] | < .001 | .218 | .040 | [.139, .297] | < .001 |
| GST autoregressive | T2–T3 | .245 | .048 | [.151, .338] | < .001 | .232 | .045 | [.144, .319] | < .001 |
| GST autoregressive | T3–T4 | .226 | .042 | [.143, .308] | < .001 | .235 | .044 | [.149, .320] | < .001 |
| CQ → GST | T1–T2 | −.127 | .026 | [−.178, −.077] | < .001 | −.197 | .039 | [−.274, −.120] | < .001 |
| CQ → GST | T2–T3 | −.090 | .027 | [−.143, −.037] | = .001 | −.133 | .040 | [−.212, −.055] | = .001 |
| CQ → GST | T3–T4 | −.101 | .027 | [−.155, −.047] | < .001 | −.151 | .041 | [−.231, −.071] | < .001 |
| GST → CQ | T1–T2 | .019 | .060 | [−.098, .137] | = .746 | .013 | .040 | [−.065, .091] | = .746 |
| GST → CQ | T2–T3 | .001 | .067 | [−.129, .132] | = .986 | .001 | .044 | [−.085, .087] | = .986 |
| GST → CQ | T3–T4 | .045 | .059 | [−.070, .160] | = .445 | .030 | .039 | [−.047, .107] | = .444 |

*Note.* RI-CLPM = random-intercept cross-lagged panel model. β values are STDYX standardized estimates from the fully unconstrained model.

# **Table S5. Formal tests of temporal stationarity**

| **Model** | **χ²** | **df** | | **p** | **CFI** | **TLI** | **RMSEA** | **SRMR** | | **AIC** | **BIC** |
| --- | --- | --- | --- | --- | --- | --- | --- | --- | --- | --- | --- |
| M1: all lagged paths free | 27.689 | 9 | | = .001 | .993 | .978 | .039 | .022 | | 31,566.922 | 31,749.865 |
| M2a: autoregressive families equal | 28.412 | 13 | | = .008 | .994 | .988 | .029 | .022 | | 31,559.444 | 31,721.479 |
| M2b: M2a + CQ → GST equal | 29.428 | 15 | | = .014 | .995 | .990 | .026 | .023 | | 31,556.624 | 31,708.206 |
| M2c: all four path families equal | 29.258 | 17 | | = .032 | .995 | .992 | .023 | .023 | | 31,552.750 | 31,693.877 |
| M2d: cross-lagged families equal; autoregressions free | 28.544 | 13 | | = .008 | .994 | .988 | .029 | .023 | | 31,560.376 | 31,722.411 |
| **Comparison** | | **Scaled Δχ²** | | | **df** | | **p** | | | **Interpretation** | |
| stationarity_m2a_vs_m1 | | .558 | | | 4 | | = .968 | | | Both autoregressive path families equal across intervals. | |
| stationarity_m2b_vs_m2a | | 1.177 | | | 2 | | = .555 | | | Adds equality of CQ-to-GST paths. | |
| stationarity_m2c_vs_m2b | | .120 | | | 2 | | = .942 | | | Adds equality of GST-to-CQ paths. | |
| stationarity_m2c_vs_m1 | | 1.869 | | | 8 | | = .985 | | | All four lagged path families equal versus free. | |
| stationarity_m2d_vs_m1 | | 1.415 | | | 4 | | = .842 | | | Both cross-lagged path families equal; autoregressions free. | |
| **Path family** | | | **Wald χ²** | | | **df** | | | **p** | | |
| CQ autoregressive equality | | | .013 | | | 2 | | | = .994 | | |
| GST autoregressive equality | | | .511 | | | 2 | | | = .775 | | |
| CQ-to-GST equality | | | 1.253 | | | 2 | | | = .534 | | |
| GST-to-CQ equality | | | .274 | | | 2 | | | = .872 | | |
| **T2–T3 contrast** | | **Difference** | | | **SE** | | **95% CI** | | | **p** | |
| CQ-to-GST T2-T3 minus T1-T2 | | .037 | | | .035 | | [−.031, .106] | | | = .286 | |
| CQ-to-GST T2-T3 minus T3-T4 | | .011 | | | .035 | | [−.058, .080] | | | = .760 | |
| GST-to-CQ T2-T3 minus T1-T2 | | −.018 | | | .081 | | [−.178, .141] | | | = .822 | |
| GST-to-CQ T2-T3 minus T3-T4 | | −.044 | | | .084 | | [−.209, .121] | | | = .603 | |
| CQ autoregression T2-T3 minus T1-T2 | | .002 | | | .043 | | [−.082, .086] | | | = .963 | |
| CQ autoregression T2-T3 minus T3-T4 | | −.004 | | | .048 | | [−.099, .091] | | | = .933 | |
| GST autoregression T2-T3 minus T1-T2 | | .033 | | | .047 | | [−.059, .126] | | | = .478 | |
| GST autoregression T2-T3 minus T3-T4 | | .019 | | | .046 | | [−.072, .110] | | | = .682 | |

*Note.* Satorra–Bentler scaled difference tests were used for nested MLR models. The full equality model versus the free model yielded scaled Δχ²(8) = 1.869, p = .985. No summer-spanning T2–T3 contrast was statistically significant.

# **Table S6. Direct comparisons of the two cross-lagged directions**

| **Interval** | **CQ → GST minus GST → CQ** | | **SE** | **95% CI** | **Raw p** | | **Holm-adjusted p** |
| --- | --- | --- | --- | --- | --- | --- | --- |
| T1–T2 | −.201 | | .051 | [−.300, −.102] | < .001 | | < .001 |
| T2–T3 | −.134 | | .055 | [−.241, −.027] | = .014 | | = .014 |
| T3–T4 | −.179 | | .055 | [−.287, −.072] | = .001 | | = .002 |
| Average | −.171 | | .026 | [−.222, −.121] | < .001 | | — |
| **Test** | | **Wald χ²** | | **df** | | **p** | |
| Joint equality of the two directions across three intervals | | 44.574 | | 3 | | < .001 | |

*Note.* The variables were placed on frozen common scales using one pooled mean and standard deviation per construct. Negative differences indicate a more negative CQ-to-GST path. These tests concern fitted prospective associations, not causal asymmetry.

# **Table S7. Multiple-indicator RI-CLPM results for the full four-item CQ measure**

| **Model** | **N** | | **χ²** | | **df** | | **p** | | **CFI** | | **TLI** | | **RMSEA** | | **SRMR** |
| --- | --- | --- | --- | --- | --- | --- | --- | --- | --- | --- | --- | --- | --- | --- | --- |
| mi_riclpm_free | 1376 | | 996.718 | | 871 | | = .002 | | .997 | | .997 | | .010 | | .023 |
| mi_riclpm_equal | 1376 | | 998.784 | | 879 | | = .003 | | .997 | | .997 | | .010 | | .024 |
| **Path** | | **Interval** | | **b** | | **SE** | | **β** | | **SE** | | **95% CI for β** | | **p** | |
| CQ autoregressive | | T1–T2 | | .286 | | .053 | | .283 | | .051 | | [.183, .383] | | < .001 | |
| GST → CQ | | T1–T2 | | .021 | | .066 | | .015 | | .046 | | [−.076, .105] | | = .752 | |
| CQ → GST | | T1–T2 | | −.159 | | .032 | | −.232 | | .046 | | [−.321, −.142] | | < .001 | |
| GST autoregressive | | T1–T2 | | .247 | | .046 | | .254 | | .045 | | [.165, .343] | | < .001 | |
| CQ autoregressive | | T2–T3 | | .281 | | .055 | | .293 | | .056 | | [.184, .402] | | < .001 | |
| GST → CQ | | T2–T3 | | .002 | | .075 | | .001 | | .053 | | [−.103, .105] | | = .983 | |
| CQ → GST | | T2–T3 | | −.122 | | .033 | | −.170 | | .046 | | [−.260, −.080] | | < .001 | |
| GST autoregressive | | T2–T3 | | .297 | | .054 | | .280 | | .050 | | [.182, .378] | | < .001 | |
| CQ autoregressive | | T3–T4 | | .292 | | .051 | | .279 | | .049 | | [.184, .375] | | < .001 | |
| GST → CQ | | T3–T4 | | .048 | | .063 | | .034 | | .045 | | [−.054, .123] | | = .444 | |
| CQ → GST | | T3–T4 | | −.128 | | .035 | | −.179 | | .048 | | [−.273, −.085] | | < .001 | |
| GST autoregressive | | T3–T4 | | .270 | | .047 | | .283 | | .049 | | [.187, .378] | | < .001 | |

*Note.* The model used 44 item-by-wave indicators: 16 CQ indicators and 28 GST indicators. No item parcels were used. The free and equal structural models were compatible, scaled Δχ²(8) = 1.829, p = .986.

# **Table S8. Equality-item-excluded measurement and structural sensitivity analyses**

**Panel A. Longitudinal measurement invariance of the three-item CQ measure**

| **Model** | **χ²** | **df** | **p** | **CFI** | **TLI** | **RMSEA** | **90% CI** | **SRMR** |
| --- | --- | --- | --- | --- | --- | --- | --- | --- |
| Configural | 26.665 | 30 | = .641 | 1.000 | 1.001 | .000 | [.000, .017] | .010 |
| Metric | 30.294 | 36 | = .736 | 1.000 | 1.001 | .000 | [.000, .014] | .012 |
| Scalar | 33.450 | 42 | = .824 | 1.000 | 1.002 | .000 | [.000, .012] | .013 |

**Panel B. Model fit for the score-based and 40-indicator RI-CLPMs**

| **Model** | **N** | **χ²** | **df** | **p** | **CFI** | **TLI** | **RMSEA** | **SRMR** |
| --- | --- | --- | --- | --- | --- | --- | --- | --- |
| Score model, free paths | 1376 | 24.229 | 9 | = .004 | .994 | .981 | .035 | .021 |
| Score model, equal paths | 1376 | 27.223 | 17 | = .055 | .996 | .993 | .021 | .024 |
| 40-indicator model, free paths | 1376 | 806.228 | 709 | = .006 | .997 | .997 | .010 | .023 |
| 40-indicator model, equal paths | 1376 | 809.974 | 717 | = .009 | .997 | .997 | .010 | .024 |

**Panel C. Freely estimated cross-lagged paths**

| **Model** | **Path** | **Interval** | **b** | **SE** | **β** | **SE** | **95% CI for β** | **p** |
| --- | --- | --- | --- | --- | --- | --- | --- | --- |
| Score model | CQ → GST | T1–T2 | −.127 | .025 | −.199 | .039 | [−.275, −.122] | < .001 |
| Score model | CQ → GST | T2–T3 | −.092 | .027 | −.140 | .040 | [−.219, −.060] | = .001 |
| Score model | CQ → GST | T3–T4 | −.083 | .027 | −.127 | .041 | [−.207, −.046] | = .002 |
| Score model | GST → CQ | T1–T2 | .011 | .062 | .007 | .040 | [−.072, .085] | = .866 |
| Score model | GST → CQ | T2–T3 | −.017 | .067 | −.011 | .044 | [−.096, .074] | = .801 |
| Score model | GST → CQ | T3–T4 | .048 | .060 | .032 | .040 | [−.046, .110] | = .424 |
| 40-indicator model | GST → CQ | T1–T2 | .038 | .068 | .026 | .047 | [−.067, .120] | = .577 |
| 40-indicator model | CQ → GST | T1–T2 | −.165 | .032 | −.240 | .046 | [−.330, −.150] | < .001 |
| 40-indicator model | GST → CQ | T2–T3 | .004 | .076 | .003 | .053 | [−.102, .107] | = .957 |
| 40-indicator model | CQ → GST | T2–T3 | −.124 | .034 | −.173 | .048 | [−.267, −.080] | < .001 |
| 40-indicator model | GST → CQ | T3–T4 | .073 | .065 | .053 | .046 | [−.038, .144] | = .258 |
| 40-indicator model | CQ → GST | T3–T4 | −.108 | .036 | −.151 | .050 | [−.248, −.053] | = .002 |

*Note.* CQ3 (unequal–equal) was deleted at all four waves. The three-item CQ retained full longitudinal scalar invariance. The score-model equality test was scaled Δχ²(8) = 3.166, p = .924; the 40-indicator equality test was scaled Δχ²(8) = 3.501, p = .899. The 40-indicator model used 12 CQ and 28 GST item-by-wave indicators and no parcels.

# **Table S9. Exhaustive leave-one-indicator-out analyses**

| **Deleted indicator** | **Path** | **Interval** | **β** | **SE** | **95% CI** | **p** |
| --- | --- | --- | --- | --- | --- | --- |
| Deleted CQ1: distant–close | CQ → GST | T1–T2 | −.176 | .040 | [−0.254, −0.097] | < .001 |
| Deleted CQ1: distant–close | CQ → GST | T2–T3 | −.132 | .040 | [−0.211, −0.052] | = .001 |
| Deleted CQ1: distant–close | CQ → GST | T3–T4 | −.157 | .042 | [−0.239, −0.075] | < .001 |
| Deleted CQ1: distant–close | GST → CQ | T1–T2 | −.008 | .041 | [−0.087, 0.072] | = .849 |
| Deleted CQ1: distant–close | GST → CQ | T2–T3 | .015 | .045 | [−0.074, 0.103] | = .748 |
| Deleted CQ1: distant–close | GST → CQ | T3–T4 | .023 | .040 | [−0.056, 0.101] | = .572 |
| Deleted CQ2: competitive–cooperative | CQ → GST | T1–T2 | −.176 | .040 | [−0.253, −0.098] | < .001 |
| Deleted CQ2: competitive–cooperative | CQ → GST | T2–T3 | −.129 | .041 | [−0.209, −0.049] | = .001 |
| Deleted CQ2: competitive–cooperative | CQ → GST | T3–T4 | −.143 | .041 | [−0.223, −0.063] | < .001 |
| Deleted CQ2: competitive–cooperative | GST → CQ | T1–T2 | .014 | .040 | [−0.065, 0.092] | = .732 |
| Deleted CQ2: competitive–cooperative | GST → CQ | T2–T3 | −.012 | .044 | [−0.098, 0.075] | = .794 |
| Deleted CQ2: competitive–cooperative | GST → CQ | T3–T4 | .036 | .040 | [−0.043, 0.115] | = .371 |
| Deleted CQ3: unequal–equal | CQ → GST | T1–T2 | −.199 | .039 | [−0.275, −0.122] | < .001 |
| Deleted CQ3: unequal–equal | CQ → GST | T2–T3 | −.140 | .040 | [−0.219, −0.060] | = .001 |
| Deleted CQ3: unequal–equal | CQ → GST | T3–T4 | −.127 | .041 | [−0.207, −0.046] | = .002 |
| Deleted CQ3: unequal–equal | GST → CQ | T1–T2 | .007 | .040 | [−0.072, 0.085] | = .866 |
| Deleted CQ3: unequal–equal | GST → CQ | T2–T3 | −.011 | .044 | [−0.096, 0.074] | = .801 |
| Deleted CQ3: unequal–equal | GST → CQ | T3–T4 | .032 | .040 | [−0.046, 0.110] | = .424 |
| Deleted CQ4: unpleasant–pleasant | CQ → GST | T1–T2 | −.183 | .040 | [−0.261, −0.104] | < .001 |
| Deleted CQ4: unpleasant–pleasant | CQ → GST | T2–T3 | −.146 | .041 | [−0.226, −0.065] | < .001 |
| Deleted CQ4: unpleasant–pleasant | CQ → GST | T3–T4 | −.147 | .041 | [−0.228, −0.066] | < .001 |
| Deleted CQ4: unpleasant–pleasant | GST → CQ | T1–T2 | −.009 | .042 | [−0.090, 0.073] | = .837 |
| Deleted CQ4: unpleasant–pleasant | GST → CQ | T2–T3 | .013 | .046 | [−0.078, 0.103] | = .780 |
| Deleted CQ4: unpleasant–pleasant | GST → CQ | T3–T4 | .014 | .040 | [−0.065, 0.093] | = .730 |

*Note.* Each scenario deleted the same CQ indicator at all four waves and recalculated CQ from the three retained items. All CQ-to-GST paths remained negative and statistically significant; all reverse paths remained nonsignificant.

# **Table S10. Construct-distinctiveness analyses**

| **Scope** | **Model** | **χ²** | **df** | **p** | **CFI** | **TLI** | **RMSEA** | **SRMR** |
| --- | --- | --- | --- | --- | --- | --- | --- | --- |
| Longitudinal | One factor | 11530.252 | 890 | < .001 | .729 | .712 | .093 | .143 |
| Longitudinal | Correlated two factors | 973.120 | 862 | = .005 | .997 | .997 | .010 | .019 |
| Wave 1 | One factor | 3149.443 | 44 | < .001 | .658 | .572 | .226 | .187 |
| Wave 1 | Correlated two factors | 61.089 | 43 | = .036 | .998 | .997 | .017 | .014 |
| Wave 2 | One factor | 2830.562 | 44 | < .001 | .665 | .581 | .221 | .183 |
| Wave 2 | Correlated two factors | 62.520 | 43 | = .027 | .998 | .997 | .019 | .013 |
| Wave 3 | One factor | 2634.925 | 44 | < .001 | .684 | .605 | .219 | .183 |
| Wave 3 | Correlated two factors | 48.259 | 43 | = .269 | .999 | .999 | .010 | .012 |
| Wave 4 | One factor | 2873.566 | 44 | < .001 | .655 | .569 | .231 | .191 |
| Wave 4 | Correlated two factors | 62.535 | 43 | = .027 | .998 | .997 | .019 | .017 |

*Note.* The longitudinal correlated two-factor model fit well and strongly outperformed the one-factor model, scaled Δχ²(28) = 8654.008, p < .001. Wave-specific comparisons likewise favored two factors at every wave.

# **Table S11. Attrition and missing-data sensitivity analyses**

| **Model** | **N** | | **χ²** | | **df** | | **p** | **CFI** | | **TLI** | | **RMSEA** | | **SRMR** | |
| --- | --- | --- | --- | --- | --- | --- | --- | --- | --- | --- | --- | --- | --- | --- | --- |
| Complete cases, free paths | 1054 | | 26.754 | | 9 | | = .002 | .992 | | .976 | | .043 | | .023 | |
| Complete cases, equal paths | 1054 | | 28.823 | | 17 | | = .036 | .995 | | .992 | | .026 | | .025 | |
| Raw IPW, equal paths | 1054 | | 28.868 | | 17 | | = .036 | .995 | | .991 | | .026 | | .025 | |
| Truncated IPW, equal paths | 1054 | | 28.891 | | 17 | | = .035 | .995 | | .991 | | .026 | | .025 | |
| Attrition-pattern model | 1376 | | 36.298 | | 21 | | = .020 | .994 | | .990 | | .023 | | .023 | |
| **Model** | | **Path** | | **Interval** | | **b** | | | **β** | | **95% CI for β** | | **p** | |  |
| Complete cases, equal paths | | CQ → GST | | T1–T2 | | −.096 | | | −.155 | | [−.213, −.097] | | < .001 | |  |
| Complete cases, equal paths | | CQ → GST | | T2–T3 | | −.096 | | | −.148 | | [−.202, −.094] | | < .001 | |  |
| Complete cases, equal paths | | CQ → GST | | T3–T4 | | −.096 | | | −.147 | | [−.201, −.093] | | < .001 | |  |
| Complete cases, equal paths | | GST → CQ | | T1–T2 | | .036 | | | .024 | | [−.031, .079] | | = .393 | |  |
| Complete cases, equal paths | | GST → CQ | | T2–T3 | | .036 | | | .023 | | [−.029, .075] | | = .390 | |  |
| Complete cases, equal paths | | GST → CQ | | T3–T4 | | .036 | | | .024 | | [−.030, .078] | | = .391 | |  |
| Raw IPW, equal paths | | CQ → GST | | T1–T2 | | −.093 | | | −.151 | | [−.209, −.093] | | < .001 | |  |
| Raw IPW, equal paths | | CQ → GST | | T2–T3 | | −.093 | | | −.143 | | [−.197, −.089] | | < .001 | |  |
| Raw IPW, equal paths | | CQ → GST | | T3–T4 | | −.093 | | | −.143 | | [−.196, −.089] | | < .001 | |  |
| Raw IPW, equal paths | | GST → CQ | | T1–T2 | | .043 | | | .028 | | [−.027, .083] | | = .317 | |  |
| Raw IPW, equal paths | | GST → CQ | | T2–T3 | | .043 | | | .027 | | [−.025, .079] | | = .314 | |  |
| Raw IPW, equal paths | | GST → CQ | | T3–T4 | | .043 | | | .028 | | [−.026, .082] | | = .314 | |  |
| Truncated IPW, equal paths | | CQ → GST | | T1–T2 | | −.094 | | | −.151 | | [−.209, −.093] | | < .001 | |  |
| Truncated IPW, equal paths | | CQ → GST | | T2–T3 | | −.094 | | | −.144 | | [−.197, −.090] | | < .001 | |  |
| Truncated IPW, equal paths | | CQ → GST | | T3–T4 | | −.094 | | | −.143 | | [−.197, −.089] | | < .001 | |  |
| Truncated IPW, equal paths | | GST → CQ | | T1–T2 | | .042 | | | .028 | | [−.027, .083] | | = .320 | |  |
| Truncated IPW, equal paths | | GST → CQ | | T2–T3 | | .042 | | | .027 | | [−.026, .079] | | = .316 | |  |
| Truncated IPW, equal paths | | GST → CQ | | T3–T4 | | .042 | | | .028 | | [−.027, .082] | | = .317 | |  |
| Attrition-pattern model | | CQ → GST | | T1–T2 | | −.105 | | | −.163 | | [−.216, −.110] | | < .001 | |  |
| Attrition-pattern model | | CQ → GST | | T2–T3 | | −.105 | | | −.156 | | [−.206, −.106] | | < .001 | |  |
| Attrition-pattern model | | CQ → GST | | T3–T4 | | −.105 | | | −.157 | | [−.207, −.106] | | < .001 | |  |
| Attrition-pattern model | | GST → CQ | | T1–T2 | | .023 | | | .015 | | [−.035, .065] | | = .558 | |  |
| Attrition-pattern model | | GST → CQ | | T2–T3 | | .023 | | | .015 | | [−.035, .065] | | = .557 | |  |
| Attrition-pattern model | | GST → CQ | | T3–T4 | | .023 | | | .015 | | [−.035, .066] | | = .557 | |  |

*Note.* Strict complete cases required all eight CQ/GST wave scores (n = 1,054). Inverse-probability weights were estimated from observed baseline predictors; truncated weights used the 1st and 99th percentiles. These checks do not rule out missingness related to unobserved values.

# **Table S12. Covariate-adjusted sensitivity analysis**

| **Path** | **Interval** | **Unadjusted β** | **Adjusted β** | **Δβ** |
| --- | --- | --- | --- | --- |
| CQ → GST | T1–T2 | −.165 | −.166 | −.001 |
| CQ → GST | T2–T3 | −.158 | −.158 | .000 |
| CQ → GST | T3–T4 | −.158 | −.159 | −.001 |
| GST → CQ | T1–T2 | .015 | .014 | −.001 |
| GST → CQ | T2–T3 | .015 | .014 | −.001 |
| GST → CQ | T3–T4 | .015 | .014 | −.001 |

*Note.* The adjusted RI-CLPM included sex, residence, sibling configuration, and highest parental education as time-invariant predictors of RI-CQ, RI-GST, W-CQ1, and W-GST1. Boys, urban residence, only-child status, and junior high school or below were the reference categories. The adjusted model fit was χ²(41) = 51.483, p = .126, CFI = .996, TLI = .993, RMSEA = .014, and SRMR = .019.

# **Table S13. Measurement invariance of CQ and GST across boys and girls**

Panel A. Cross-sex longitudinal measurement-invariance models

| **Construct** | **Model** | **χ²** | **df** | **p** | **CFI** | **TLI** | **RMSEA [90% CI]** | **SRMR** |
| --- | --- | --- | --- | --- | --- | --- | --- | --- |
| CQ | Configural across sex and time | 136.307 | 148 | .745 | 1.000 | 1.001 | .000 [.000, .014] | .017 |
| CQ | Longitudinal metric within sex | 152.927 | 166 | .758 | 1.000 | 1.001 | .000 [.000, .013] | .021 |
| CQ | Longitudinal scalar within sex | 180.116 | 184 | .567 | 1.000 | 1.000 | .000 [.000, .016] | .023 |
| CQ | Cross-sex metric | 182.460 | 187 | .580 | 1.000 | 1.000 | .000 [.000, .015] | .023 |
| CQ | Cross-sex scalar | 184.040 | 190 | .608 | 1.000 | 1.001 | .000 [.000, .015] | .023 |
| GST | Configural across sex and time | 682.963 | 604 | .014 | .997 | .996 | .014 [.007, .019] | .022 |
| GST | Longitudinal metric within sex | 727.151 | 640 | .009 | .997 | .996 | .014 [.008, .019] | .026 |
| GST | Longitudinal scalar within sex | 756.292 | 676 | .017 | .997 | .996 | .013 [.006, .018] | .027 |
| GST | Cross-sex metric | 760.560 | 682 | .019 | .997 | .997 | .013 [.006, .018] | .027 |
| GST | Cross-sex scalar | 764.358 | 688 | .023 | .997 | .997 | .013 [.005, .018] | .027 |

Panel B. MLR-scaled tests of cross-sex equality constraints

| **Construct** | **Constraint added** | **Scaled Δχ²** | **df** | **p** | **ΔCFI** | **ΔRMSEA** | **ΔSRMR** |
| --- | --- | --- | --- | --- | --- | --- | --- |
| CQ | Equal loadings across sex | 2.491 | 3 | .477 | .000 | .000 | .000 |
| CQ | Equal intercepts across sex | 1.627 | 3 | .653 | .000 | .000 | .000 |
| GST | Equal loadings across sex | 5.469 | 6 | .485 | .000 | .000 | .000 |
| GST | Equal intercepts across sex | 4.477 | 6 | .612 | .000 | .000 | .000 |

Note. Models retained correlated residuals for the same item across waves and used MLR with all available data. The sequence progressed from a common configural form to longitudinal metric and scalar invariance within each sex, followed by equality of loadings and intercepts across sex. Full metric and scalar invariance was supported for both CQ and GST under the complete set of cross-sex loading and intercept constraints.

# **Table S14. Score-based multigroup RI-CLPM results and sex-group equality tests**

Panel A. Model fit

| **Model** | **N** | **χ²** | **df** | **p** | **CFI** | **TLI** | **RMSEA [90% CI]** | **SRMR** |
| --- | --- | --- | --- | --- | --- | --- | --- | --- |
| Free paths across time and sex | 1,376 | 34.020 | 18 | .013 | .994 | .981 | .036 [.016, .054] | .024 |

Panel B. Freely estimated focal cross-lagged paths

| **Group** | **Path** | **Interval** | **b** | **SE** | **β** | **SE** | **95% CI for β** | **p** |
| --- | --- | --- | --- | --- | --- | --- | --- | --- |
| Boys | CQ → GST | T1–T2 | −.100 | .035 | −.162 | .055 | [−.270, −.054] | .003 |
| Boys | CQ → GST | T2–T3 | −.088 | .037 | −.142 | .059 | [−.258, −.025] | .017 |
| Boys | CQ → GST | T3–T4 | −.088 | .037 | −.138 | .059 | [−.253, −.023] | .019 |
| Boys | GST → CQ | T1–T2 | .108 | .080 | .071 | .052 | [−.032, .173] | .178 |
| Boys | GST → CQ | T2–T3 | .013 | .093 | .008 | .058 | [−.106, .123] | .887 |
| Boys | GST → CQ | T3–T4 | −.009 | .092 | −.005 | .057 | [−.117, .106] | .924 |
| Girls | CQ → GST | T1–T2 | −.149 | .039 | −.220 | .057 | [−.331, −.109] | < .001 |
| Girls | CQ → GST | T2–T3 | −.087 | .039 | −.119 | .054 | [−.224, −.013] | .027 |
| Girls | CQ → GST | T3–T4 | −.126 | .041 | −.179 | .057 | [−.292, −.067] | .002 |
| Girls | GST → CQ | T1–T2 | −.062 | .088 | −.042 | .059 | [−.158, .075] | .483 |
| Girls | GST → CQ | T2–T3 | −.005 | .096 | −.004 | .067 | [−.135, .127] | .956 |
| Girls | GST → CQ | T3–T4 | .077 | .077 | .056 | .055 | [−.053, .164] | .315 |

Panel C. Formal sex-group and stationarity tests

| **Test** | **χ² / scaled Δχ²** | **df** | **p** | **Interpretation** |
| --- | --- | --- | --- | --- |
| Joint sex difference: CQ → GST | 1.168 | 3 | .761 | No statistically detectable difference |
| Joint sex difference: GST → CQ | 2.704 | 3 | .440 | No statistically detectable difference |
| Joint sex difference: CQ autoregression | 1.095 | 3 | .778 | No statistically detectable difference |
| Joint sex difference: GST autoregression | 7.045 | 3 | .071 | No statistically detectable difference |
| Within-sex stationarity: R1 vs. R0 | 10.190 | 16 | .857 | Temporal equality compatible with data |
| Cross-sex equality: CQ → GST | .388 | 1 | .533 | Equality constraint supported |
| Cross-sex equality: GST → CQ | .048 | 1 | .827 | Equality constraint supported |
| Cross-sex equality: both cross-lagged families | .242 | 2 | .886 | Equality constraints supported |
| Cross-sex equality: all lagged families | 2.091 | 4 | .719 | Equality constraints supported |
| Random-intercept covariance equality | .012 | 1 | .912 | No statistically detectable difference |

Note. RI-CLPM = random-intercept cross-lagged panel model. Equality and Wald tests were applied to unstandardized coefficients; standardized coefficients are reported descriptively. None of the six interval-specific sex contrasts was significant after Holm adjustment. The random-intercept correlations were r = −.290 for both boys and girls. Nonsignificant difference tests indicate that no sex-group moderation was detected; they do not establish exact equivalence.

# **Table S15. Multiple-indicator multigroup RI-CLPM sensitivity analysis**

Panel A. Model fit

| **Model** | **N** | **χ²** | **df** | **p** | **CFI** | **TLI** | **RMSEA [90% CI]** | **SRMR** |
| --- | --- | --- | --- | --- | --- | --- | --- | --- |
| Cross-lagged paths free across sex | 1,376 | 1976.425 | 1760 | < .001 | .995 | .994 | .013 [.010, .016] | .031 |
| CQ → GST paths equal across sex | 1,376 | 1977.888 | 1763 | < .001 | .995 | .994 | .013 [.010, .016] | .031 |
| GST → CQ paths equal across sex | 1,376 | 1978.841 | 1763 | < .001 | .995 | .994 | .013 [.010, .016] | .031 |
| Both cross-lagged families equal across sex | 1,376 | 1980.471 | 1766 | < .001 | .995 | .994 | .013 [.009, .016] | .032 |

Panel B. MLR-scaled cross-sex equality tests

| **Constraint** | **Scaled Δχ²** | **df** | **p** | **ΔCFI** | **ΔRMSEA** | **ΔSRMR** |
| --- | --- | --- | --- | --- | --- | --- |
| CQ → GST paths equal across sex | 1.225 | 3 | .747 | .000 | .000 | .000 |
| GST → CQ paths equal across sex | 2.416 | 3 | .491 | .000 | .000 | .000 |
| Both cross-lagged families equal across sex | 4.188 | 6 | .651 | .000 | .000 | .001 |

Panel C. Freely estimated focal paths

| **Group** | **Path** | **Interval** | **b** | **SE** | **β** | **SE** | **95% CI for β** | **p** |
| --- | --- | --- | --- | --- | --- | --- | --- | --- |
| Boys | CQ → GST | T1–T2 | −.123 | .042 | −.192 | .064 | [−.318, −.067] | .003 |
| Boys | CQ → GST | T2–T3 | −.127 | .045 | −.194 | .069 | [−.328, −.059] | .005 |
| Boys | CQ → GST | T3–T4 | −.109 | .047 | −.163 | .071 | [−.301, −.024] | .021 |
| Boys | GST → CQ | T1–T2 | .109 | .089 | .074 | .060 | [−.045, .192] | .221 |
| Boys | GST → CQ | T2–T3 | .023 | .109 | .015 | .071 | [−.125, .155] | .832 |
| Boys | GST → CQ | T3–T4 | −.006 | .100 | −.004 | .065 | [−.132, .124] | .948 |
| Girls | CQ → GST | T1–T2 | −.189 | .050 | −.256 | .067 | [−.386, −.125] | < .001 |
| Girls | CQ → GST | T2–T3 | −.108 | .048 | −.137 | .061 | [−.257, −.017] | .026 |
| Girls | CQ → GST | T3–T4 | −.160 | .052 | −.210 | .066 | [−.339, −.081] | .001 |
| Girls | GST → CQ | T1–T2 | −.062 | .097 | −.044 | .070 | [−.182, .093] | .526 |
| Girls | GST → CQ | T2–T3 | −.009 | .107 | −.007 | .081 | [−.166, .152] | .935 |
| Girls | GST → CQ | T3–T4 | .087 | .082 | .068 | .063 | [−.056, .192] | .285 |

Note. The model used 44 item-by-wave indicators: 16 CQ and 28 GST indicators; no item parcels were used. All models terminated normally. The item-level analyses corroborated the score-based finding that no statistically detectable sex-group difference emerged in either cross-lagged direction.

# **Supplementary references**

Islam, M. R., & Hewstone, M. (1993). Dimensions of contact as predictors of intergroup anxiety, perceived out-group variability, and out-group attitude: An integrative model. Personality and Social Psychology Bulletin, 19(6), 700–710. https://doi.org/10.1177/0146167293196005

Moreau, C., Li, M., Ahmed, S., Zuo, X., & Cislaghi, B. (2021). Assessing the spectrum of gender norms perceptions in early adolescence: A cross-cultural analysis of the Global Early Adolescent Study. Journal of Adolescent Health, 69(1), S16–S22. https://doi.org/10.1016/j.jadohealth.2021.03.010

Yu, C., Zuo, X., Lian, Q., Zhong, X., Fang, Y., Lou, C., et al. (2022). Comparing the perceptions of gender norms among adolescents with different sibling contexts in Shanghai, China. Children, 9(9), Article 1281. https://doi.org/10.3390/children9091281

Yuan, X., Lei, T., Su, Y., Li, X., & Zhu, L. (2024). Longitudinal associations between intergroup contact and intergroup trust among adolescents in ethnic regions of China. Scientific Reports, 14, Article 15942. https://doi.org/10.1038/s41598-024-66646-1
